# Consumption and Greenhouse Gas Emissions Impacts of Population‐Wide Adoption of Dietary Guidelines in China

**DOI:** 10.1111/nbu.70008

**Published:** 2025-04-18

**Authors:** Sabina Crowe, Rosemary Green, Christian Reynolds, Bhavani Shankar

**Affiliations:** ^1^ Northeastern University – London London UK; ^2^ London School of Hygiene & Tropical Medicine London UK; ^3^ City St Georges, University of London London UK; ^4^ University of Sheffield Sheffield UK

**Keywords:** diet, dietary guidelines, environmental impact, foods, greenhouse gas emissions, People's Republic of China

## Abstract

This paper uses an optimisation model to quantify the necessary food consumption adjustments for Chinese diets to fulfil the requirements in the health‐based Chinese Dietary Guidelines (CDG) or WHO dietary guidelines. We further aim to determine whether adopting these guidelines could lead to lower levels of greenhouse gas emissions (GHGE) while maintaining diet affordability. Modelling outcomes under the CDG and WHO scenarios differ significantly from nutritional, GHGE and diet affordability perspectives: relative to observed eating patterns, diets following the WHO guidelines are equally emissions intensive, while diets consistent with the CDG recommendations are less sustainable. Further optimisations imposing significant reductions in GHGE indicate important environmental and nutritional co‐benefits can be achieved through the WHO guidelines, while maintaining diet affordability. In the WHO scenario, the maximum diet‐related GHGE reduction policymakers could aim for is 30%, since above this threshold, recommended diets would deviate considerably from observed patterns. The CDG model with a 20% emissions reduction does not converge for 64% of the initial data set, casting doubt on the affordability and compatibility of the CDG with China's decarbonisation goal. We recommend that future versions of the CDG be reformulated to closer align with WHO advice and explicitly include environmental considerations.

## 1 Introduction

Food is essential for human survival, yet its production is the most impactful human activity on the environment, threatening the very ecosystems we depend on (Freidberg 2016). Several studies (Barilla 2021; Sachs et al. 2020; Willett et al. 2019; Pradyumna et al. 2019; Béné et al. 2019) have emphasised the negative environmental impacts of modern food systems and discussed their substantial implications for our collective well‐being. Some authors have shown that eating according to health‐focused dietary guidelines (Springmann et al. 2018; Behrens et al. 2017; Luna‐Cortés 2017; Green et al. 2015) can lead to a reduction in greenhouse gas emissions (GHGE), whereas others (Springmann et al. 2020; Wang et al. 2020; Ritchie et al. 2018; van de Kamp et al. 2018) have found that, under their current formulations, most national dietary guidelines are incompatible with long‐term climate mitigation goals.

China is the world's largest emitter of greenhouse gases (Climate Watch 2024), and its rapid rates of industrialisation and urbanisation over the past few decades have driven an ongoing nutrition transition (Huang et al. 2021), with considerable negative implications for both the environment and public health (Popkin 2014). China now faces significant nutrition challenges, including a shift towards increased consumption of animal‐source and processed foods. This trend has contributed to rising rates of obesity and overweight, while undernutrition and micronutrient deficiencies continue to be major concerns. More than half of adults in China consume inadequate amounts of key nutrients such as thiamine, riboflavin, vitamin C and calcium (Huang et al. 2021). In addition to these micronutrient deficiencies, selenium deficiency remains a concern in certain regions, highlighting the need for dietary adjustments to address these widespread nutritional gaps (Li et al. 2014).

China plans to peak CO_2_ emissions before 2030, reduce CO_2_ emissions per unit of GDP by more than 65% compared to the 2005 level by 2030 and achieve carbon neutrality by 2060 (Climate Watch 2024). Since, in 2019, China's food system generated approximately 1.9 billion tonnes of CO_2_‐equivalent emissions, or about 13.5% of the country's total GHGE (Oxford Institute of Energy Studies 2022), measures to reduce food‐related emissions are essential to its decarbonisation strategy.

Against this background, revised food‐based Chinese Dietary Guidelines (CDG) were published in 2016 and, most recently, in 2022. The overall implications for sustainability of the CDG remain unclear: only a limited amount of quantitative information is available for China on the potential effects that compliance with the CDG could have on the intakes of specific foods and associated levels of CO_2_. For instance, Lei and Shimokawa (2020) examined the effects of the 2007 dietary guidelines, while He et al. (2019) analysed the environmental impacts of the 2016 CDG. Ritchie et al. (2018) estimated the implications of the 2016 CDG and WHO recommendations on global emissions levels but did not specifically compute the effects of the two sets of guidelines for China. Wang et al. 2020 explored the shifts in environmental footprints associated with diets adjusting in line with the minimum recommended intakes in the 2016 CDG and found a 29% decrease in the carbon footprint. However, they calculated that, if domestic consumption adjusted to meet the CDG's upper limit of recommended intake for each food group, carbon emissions would rise by 22%. None of these studies relied on optimisation models to identify suitable diets or computed the expected costs of such diets—a gap this paper fills.

The WHO guidelines were designed to fight chronic diseases, contain recommendations for specific intakes of different macro‐ and micronutrients considered to be conducive to better health (FAO/WHO 2003) and have become the basis on which national governments often develop their dietary advice. These WHO guidelines represent the gold standard of nutritional guidelines against which studies have analysed diets in a variety of countries, and their adoption has been shown (Green et al. 2015; Ritchie et al. 2018) to have the potential to reduce GHGE while enabling public health. The CDG are food‐based, specifying the quantities of each major food group to be consumed daily, but the WHO recommendations do not impose specific restrictions on food categories, except for fruits and vegetables. While the CDG are not directly based on the WHO dietary guidelines, they have undoubtedly been influenced by them and other international standards. Both the WHO and the CDG emphasise the importance of nutrient‐dense foods, advocating for a diverse diet rich in fruits, vegetables and whole grains, while also limiting the intake of sodium, sugar and processed foods. A diet that conforms to the CDG would likely align with the WHO advice in many respects, but there could be differences, particularly regarding animal protein intake. To our knowledge, no test diets have been developed to evaluate whether following the CDG results in a nutritionally balanced diet according to WHO guidelines. However, Zhu et al. (2023) noted that the CDG evolved from focusing on survival to promoting nutritional balance and disease prevention. Tools like the Chinese Healthy Eating Index (Yuan et al. 2017) assessed adherence to the 2016 CDG using *China Health and Nutrition Survey* (*CHNS*) 2011 data, while Wu et al. (2022) analysed shifts in urban and rural Chinese diets in 2019 to align with dietary recommendations. However, neither study involved experimental test diets.

The alignment, or divergence, between the two sets of guidelines presents a key area for exploration, as it offers valuable insight into how national guidelines might depart from global health goals. Given the different formats in which advice is provided, a direct comparison between the guidelines is not straightforward. Our paper addresses this challenge through a modelling exercise.

Previous studies have used diet optimisation models to assist in identifying sustainable diets (Fu et al. 2024; Gazan et al. 2018; Springmann et al. 2018; Ritchie et al. 2018; Horgan et al. 2016). Green et al. (2015) use quadratic programming (QP) optimisation to calculate UK diets compliant with WHO recommendations and consider potential substitutions between food groups by incorporating food price elasticities and expenditure shares in the objective function. The objective function represents an approximation of consumers' loss in welfare from adopting a new diet, and thus minimising it under a budget constraint ensures resulting diets are realistic and likely to be appealing to consumers. A further discussion of the diet optimisation model literature can be found in Appendix E.

The aim of this paper is to use dietary optimisation to quantify the potential impact of the adoption of dietary guidelines on GHGE in China, by modelling affordable diets that comply with either the CDG or the WHO recommendations. We adopt the QP model introduced in Green et al. (2015) that we employ in conjunction with the most recent wave of household food survey data from the *CHNS*. Essentially, we verify whether diets following the two dietary guidelines are cost‐effective and more environmentally sustainable relative to observed eating habits, or whether the guidelines should be reformulated to include additional sustainability criteria.

This paper is significant as it provides a quantification of the prospective deviations in average GHGE levels, diet costs and food consumption levels (for 19 core food groups) resulting from a generalised dietary shift in China. By incorporating food price elasticities and budget shares in our models, we account for substitutions between food groups, therefore improving on traditional QP models (see Appendix F for more details). Moreover, as far as we are aware, no previous studies of Chinese diets focused on comparing the implications of adhering to the WHO dietary guidelines with the probable outcomes of the 2016 CDG.

## 2 Data and Methods

### 2.1 Data

#### 2.1.1 Food Consumption, Socio‐economic, Price and Elasticity Data

This study employed data on 9040 adults aged 18–65 years from the 2011 *CHNS* food survey wave. The *CHNS* food consumption data were collected over three consecutive days, tracking both individual and household consumption. Household food inventory was weighed at the start and end of the survey, while individual consumption was recorded through 24‐h recalls, including food consumed away from home (CHNS 2015). In 2011, cross‐sectional data was gathered from 12 Chinese provinces and megacities, encompassing 288 communities. To our knowledge, this is the most recent publicly accessible dietary survey of the Chinese population, with the more recent CHNS 2015 food consumption data not available on the website (CHNS 2015). The *CHNS* stopped collecting the survey data after the 2015 wave.

These data were used in conjunction with the 2002 and 2004 Chinese food composition tables (FCT) which provided information on the protein, fat, carbohydrate and sodium content of each food. Data on total sugar content were extrapolated from the USDA Food Databases by matching individual foods from the Chinese FCT with foods in the USDA tables.

The foods from the FCT were initially aggregated into 106 sub‐groups and 19 overarching groups; later, the 19 food groups were aggregated into 10 overarching categories (cereals, fruits, vegetables, meat, dairy, eggs, fish, soybeans, processed foods, fats and oils). Following the steps detailed in Green et al. (2015), we computed sub‐group nutrient profiles for the five key nutrients mentioned above that, together with the average daily intake of each sub‐group, allowed us to determine a weighted average of the nutritional content for each of the 19 groups.

We used socio‐economic variables from CHNS, including age, gender and rural/urban location, collected alongside food consumption data. Total household net income information, also from the *CHNS*, tracks all income sources for each household in 2011 (CHNS 2015).

Community‐specific price data from the *CHNS* were used. For each of the 288 communities surveyed, we employed 36 food prices that we deemed the most relevant and had the most complete data. These prices were matched with individual foods pertaining to the food consumption dataset to provide a weighted average price for each of the 19 food groups.

Group‐level food price elasticities were sourced from Chen et al. (2016) and Seale (2012). Appendix A displays these values, together with the overall mean budget shares used in our models.

#### 2.1.2 Greenhouse Gas Emissions Data

Life cycle assessment (LCA) data on GHGE at the food group level were compiled from a variety of sources in China, given that the bulk of the food consumed in China in 2011 was produced domestically. Where China‐specific data were not available, extrapolations from Industrialised Asia, USA or France were conducted. When a wide range of emissions estimates for the same food item was found in the literature, we assumed a log‐normal distribution of these values, and we calculated the carbon footprint of that food item as their geometric mean, as commonly done in the literature (Vieux et al. 2012). See Appendix B for more information on the GHGE data used and their variation. Dairy product carbon footprints were calculated using milk emissions multiplied by FAO extraction rates (FAO n.d.).

Most GHGE values for China came from food production, accounting only for emissions up to the farm gate. To compute LCA estimates, we considered the carbon footprint of all stages in the food production chain: production, processing, packaging, storage, transportation, cooking and waste. Data on the emissions associated with each stage were obtained from various sources—refer to Appendix B for more information.

We calculated group‐level emissions by matching individual foods within each food group to those with available emissions values. We computed group‐level average emissions based on each food's CO_2_ footprint and its proportion in total group consumption. Refer to Appendix B for more information on the GHGE footprints of food groups.

### 2.2 Methods

#### 2.2.1 Model Specification

The two scenarios we modelled—CDG and WHO (see Table 1 for an overview of dietary restrictions in each case)—are based on the same theoretical concept, share the same objective function and a few of the constraints (e.g., cost not exceeding the net average daily income per person and ensuring non‐negative consumption of each food item—see Appendices C, D and F for all constraints). The models minimise the loss in consumer welfare (i.e., the sum of the weighted deviations between the observed and optimised diets), undernutrition, budget and environmental constraints. The objective function takes the following form:

(1) $$\min_{Q_{i}^{\prime }}\;\sum_{i=1}^{19}\;\frac{s_{i}}{\varepsilon_{i}}∙{\left(\frac{Q_{i}^{\prime }-Q_{i}}{Q_{i}}\right)}^{2},$$

where $Q_{i}^{\prime }$ and $Q_{i}$ are components of the optimised and observed diet, respectively, *s*_*i*_ is the expenditure share on food group *i*, ε_*i*_ represents the own‐price demand elasticity of food category *i* . *i* takes on a value from 1 to 19 since, for every individual in the dataset, the model is optimised across all 19 food kinds in order to ensure everybody meets all the CDG recommendations. The optimised consumption of each food group is assumed to be greater than zero in all the models. For non‐consumers of a given group, we assumed the starting consumption level to be 0.0001 g instead of an absolute 0, to ensure the model functioned. Our assumption is reasonable, as traces of many food items are often present in various dishes.

**TABLE 1:** Observed average per day intakes of key food groups and nutrients, separately by gender, together with the Chinese Dietary Guidelines (CDG) and World Health Organization (WHO) recommendations, and the average daily level of dietary‐related greenhouse gas emissions (GHGE, grams of CO_2_ eq.)

**Observed consumption versus dietary guidelines recommendations**			
	**Males**	**Females**	
Number of individuals	4214	4826	
	**Observed consumption levels**		**2016 CDG recommendations**
Total energy (kcal)	2093.65	1769.93	1600–2400
Food categories (g/day)			
Cereals (wheat, rice, corn, coarse grains, beans and tubers)	480.39 ^a^	389.74	250–400
Soybeans and nuts	59.25	53.94	25–35
Vegetables (vegetables, leafy greens, mushrooms)	296.98 ^a^	271.14	300–500
Fruit (fruit)	57.76	69.52	200–350
Meat (pork, beef, mutton and poultry)	104.11	80.61	40–75
Fish	27.65	23.73	40–75
Eggs	31.18	28.81	40–50
Dairy	25.25	31.19	300
Processed foods (sweets, savoury foods and drinks)	65.06	53.65	n/a
Fats and oils^b^	25.00	25.00	25–35
Nutrients			
Sodium chloride (mg)	2500	2105	< 6000 mg
Total sugar (g)	24.98	23.50	n/a
Added sugar (g)	4.36	4.18	< 25 g
	**Observed consumption levels**		**WHO recommendations**
Nutrients (% of daily calories)			
Fat	31.5%	32.9%	15%–30%
Protein	13.1%	12.9%	10%–15%
Carbohydrate	55%	54%	55%–75%
Total sugar^c^	4.8%	5.4%	< 10%
Sodium	998.08	841.81	< 2000 mg
Food groups (g/day)			
Fruits and vegetables	354.74	340.66	≥ 400 g
	**Diet‐related GHGE**		**WHO/CDG recommendations**
GHGE (grams of CO_2_ eq./day)	2146.38	1766.60	n/a

^a^ Figures in red indicate overconsumption, whereas blue shows underconsumption.

^b^ Due to data on fats and oil consumption being unavailable, we set the fats and oils intake equal to the lower bound of the CDG recommendations.

^c^ We consider here the stricter recommendation capping the intake of total sugar (as opposed to free sugars).

We optimised under a budget constraint that ensured the daily diet cost did not exceed the average daily net income of each consumer. We adopted this approach because imposing a constraint to limit food expenditure to 40%–45% of household income resulted in 20%–30% of households failing to meet the objective function, thus considerably narrowing the scope of the analysis. We verified that our average food expenditure shares align with values reported in the literature (e.g., Chen et al. 2016). Using the average daily income per household member makes sense, as families typically share meals and purchase food together, ensuring that non‐income‐earning members are appropriately considered. This approach is consistent with previous literature (e.g., Zuercher et al. 2011), however, it has limitations, as it oversimplifies budget allocations and does not account for other essential household expenses.

The WHO constraints model core recommendations on protein, fat, sugar and sodium intakes (see Table 1). Additional restrictions set the minimum daily fruit and vegetable intake at 400 g, limit fat energy to 15%–30% of daily calories, and set protein energy between 10% and 15%. Due to data limitations, constraints on saturated fat, polyunsaturated fats and cholesterol are excluded. Other constraints include a stricter 2 g/day sodium limit, a maximum of 10% of daily energy from total sugar and maintaining the same total energy in the optimised diet as the original to preserve consistency with the observed diet. The WHO recommends limiting free sugars to 10% of daily energy. However, due to the unavailability of free sugar data and the fact that the observed total sugar intake was ~5% of daily calories for both men and women, we opted to use total sugars instead. Appendix C lists these constraints in equation form.

The CDG model restrictions follow key recommendations from the Food Pagoda (China Nutrition Society 2016)—see Table 1. The first constraint keeps the optimised diet's total energy between 1600 and 2400 kcal, while other constraints apply the same upper or lower bounds on food groups as in the Food Pagoda. We model the extended CDG recommendation to limit added sugars to 25 g/day. Since data on added sugar were unavailable, we model a proxy of this restriction by limiting the total sugar intake from processed foods (including sweets, savoury snacks and non‐alcoholic beverages) to 25 g/day. Since processed foods contain high amounts of added sugars, it makes sense to assume their total sugar content roughly approximates their added sugar content. Appendix D lists these restrictions in equation form.

Although revised CDG were published in 2022, we used the 2016 CDG due to data availability constraints—2016 guidelines are more likely to align with food consumption patterns in place during the 2011 CHNS wave considered here. Table 2 compares the two versions of the guidelines (2016 and 2022): the main differences in 2022 consist in the more restrictive salt recommendation (from < 6 g/day to < 5 g in 2022), lower cereals and tubers recommendations (250–400 g/day to 200–300 g in 2022), and higher dairy recommendations (500 g/day in 2022). Both editions of the CDG advise consumers to eat the same amounts of total meat, including pork, beef, mutton and poultry (40–75 g/day) as well as fish (40–75 g/day).

**TABLE 2:** Chinese Dietary Guidelines—comparison between the 2016 and 2022 versions.

	Recommended daily intake	
Food/nutrient	2016 CDG	2022 CDG
Salt	**< 6 g**	**< 5 g**
Oil	25–30 g	25–30 g
Milk and dairy	**300 g**	**300–500 g**
Soybeans and nuts	25–35 g	25–35 g
Meat (such as poultry, pork and beef)	40–75 g	40–75 g
Aquatic products	40–75 g	40–75 g
Eggs	40–50 g	1 egg/day (approx. 50 g)
Vegetables	300–500 g	300–500 g
Fruits	200–350 g	200–350 g
Cereals, tubers and legumes	**250–400 g**	**200–300 g**
Water	1.5–1.7 L	1.5–1.7 L

*Note:* Differences in bold.

*Source:* China CDC (2022) and China Nutrition Society (2016).

After running models containing the above constraints only, we assessed the potential reduction in GHGE. For that purpose, a further constraint was added that progressively reduced by 5% the maximum amount of emissions allowed in each scenario. The greatest reduction in GHGE modelled for the WHO case was 40%. Since the CDG diet is much more prescriptive than its WHO counterpart, the model did not converge for higher levels of emissions reduction and usually stopped returning results after the 15% drop in emissions threshold.

## 3 Results

### 3.1 Baseline Consumption, Diet Costs and Greenhouse Gas Emissions

Table 1 shows the average intakes of key food groups and nutrients at the baseline, together with the modelled CDG and WHO recommendations. The average diet for both males and females exceeds CDG recommendations for total meat intakes as well as for soybeans and nuts. The average consumption of fruit, fish, eggs and dairy is considerably below suggested amounts for both genders, with observed dairy intakes representing less than 10% of the CDG target amount of 300 g/day. Average energy levels align with the CDG advice, while the WHO does not impose restrictions on daily kilocalorie consumption. Fat consumption exceeds WHO recommendations for both genders, and carbohydrate intake meets the lower bound of the recommendations for men but falls slightly short for women. Protein, sugar and sodium intakes are within recommended limits. We acknowledge that the mean sodium intake may appear low compared to earlier reports on salt consumption in China, especially given that condiments and salt added during cooking were included in the analysis. However, our findings are consistent with other studies that show a decline in sodium intake between 1989 and 2012 to levels significantly below the CDG recommendations (Huang et al. 2021). This apparent decrease is attributed to a rise in eating out and an increased consumption of processed and pre‐packaged foods, which complicates accurate sodium intake estimation, as not all sources are fully captured in individual reporting.

Neither guideline sets a target carbon footprint level for consumers. However, we found the average daily level of dietary‐related GHGE to be 1943.63 g of CO_2_ eq. for the whole dataset population, or 2146.38 g of CO_2_ eq. for males and 1766.6 g of CO_2_ eq. for females. This corresponds to approximately 709 kg of CO_2_/person/year, very similar to Ritchie et al. (2018)'s estimate of 757 kg of CO_2_ per capita per year for the non‐vegetarian Indian diet. Our estimates place Chinese diets well below UK diets in emissions intensity, with Scarborough et al. (2014) estimating 2624.35 kg CO_2_/person/year for high meat eaters and 2054.95 kg CO_2_/person/year for medium meat eaters in the United Kingdom.

**FIGURE 1:**
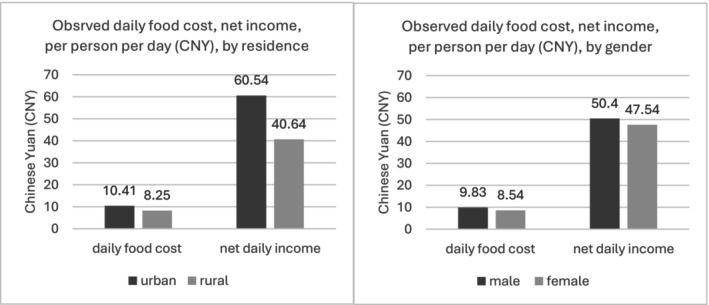
Observed, average daily food cost and net daily income per person (CNY) for urban and rural residents, males and females.

Figure 1 compares the observed average daily food cost with daily net income per person for urban and rural residents as well as for males and females. For urban consumers, the diet cost represents approximately 17.2% of daily net income, whereas for rural consumers, it rises to 20.3% of the daily net income.

### 3.2 Models Without Environmental Constraints

The CDG constraints are stringent since they stipulate specific intakes of key food groups. When adding a budget constraint limiting the daily food cost to the daily net income/person, the model does not find a solution for 7.6% of the total individuals in the original dataset. The WHO guidelines are less prescriptive, allowing the corresponding optimisation with nutritional and budget constraints to converge in 99.9% of cases. The findings below correspond to a dataset consisting of 8537 individuals for which both the CDG and WHO models converge. The analysis was conducted separately for each individual, however findings are presented at various aggregation levels.

**FIGURE 2:**
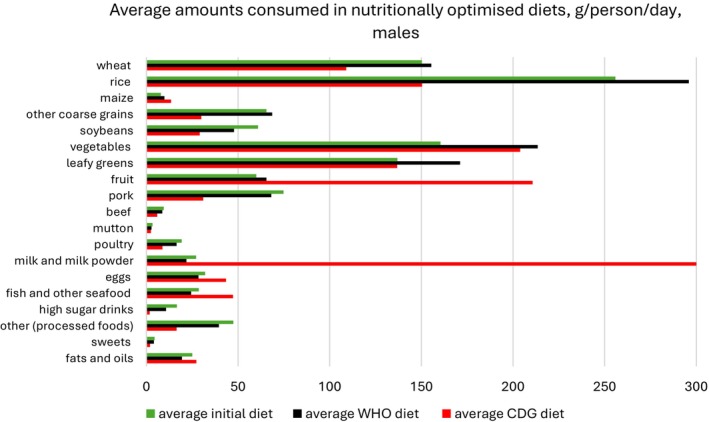
Nutritionally optimised (males) under the Chinese Dietary Guidelines (CDG) and World Health Organization (WHO) scenarios with no environmental constraints, compared with the original average diet, re‐calculated for *n* = 8537 individuals; the WHO diet was optimised for macronutrients, total sugars, salt and fruit and vegetables.

**FIGURE 3:**
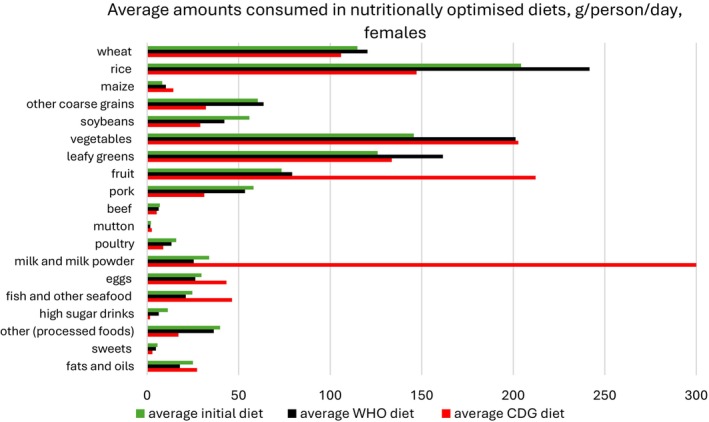
Nutritionally optimised diet (females) under the Chinese Dietary Guidelines (CDG) and World Health Organization (WHO) scenarios with no environmental constraints, compared with the original average diet, re‐calculated for *n* = 8537 individuals; the WHO diet was optimised for macronutrients, total sugars, salt and fruit and vegetables.

Figures 2 and 3 show the average diets for males and females, under the CDG and WHO scenarios without environmental constraints, and compare these with the original average diet, re‐calculated so it only includes the 8537 individuals retained in the final data set. The average WHO diet for both genders involves higher amounts of wheat, rice and coarse grains compared to the CDG and observed diets. Both optimised diets recommend higher amounts of vegetables, leafy greens and fruit relative to observed intakes for both males and females. Meanwhile, pork, beef and poultry are consumed more sparingly in the optimised diets. Mutton intakes for women rise in the CDG model but decrease for men. While milk, fish and egg consumption increase for both genders in the CDG diet, they decrease in the generated WHO diet. The quantities of processed foods, high sugar drinks and sweets experience a drop in both models and for both genders.

**FIGURE 4:**
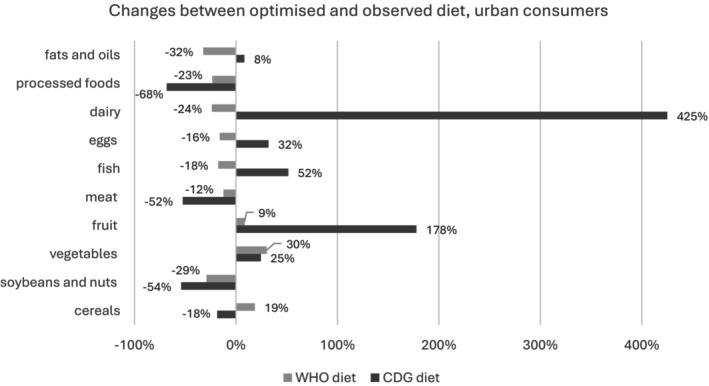
Necessary shifts (%) in daily food intakes to bring observed diets in line with dietary requirements, urban consumers.

**FIGURE 5:**
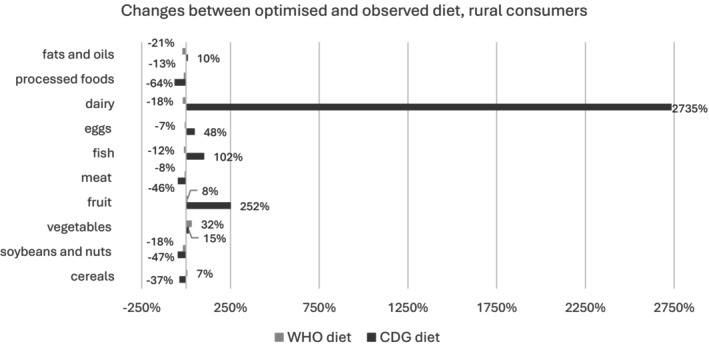
Necessary shifts (%) in daily food intakes to bring observed diets in line with dietary requirements, rural consumers.

Figures 4 and 5 present the necessary shifts in daily food intakes to bring observed diets in line with dietary requirements, separately for urban and rural consumers. Dairy consumption needs to increase by almost 3000% for rural consumers to reach the 300 g/day CDG recommended intake. Meanwhile, the WHO diet would lead to an 18% decrease in dairy consumption for rural residents, thus emphasising the considerable gap between the two sets of dietary guidelines. Fruit is another food category for which intakes would rise significantly in the CDG‐modelled diets, but only slightly in the WHO one, for both rural and urban residents.

**FIGURE 6:**
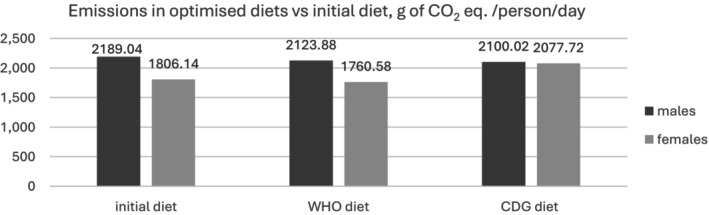
Average level of diet‐related emissions in the World Health Organization (WHO) and Chinese Dietary Guidelines (CDG) nutritionally optimised models, g of CO_2_ eq./person/day.

Figure 6 indicates the CDG diet would lead to a rise in emissions for females relative to initial levels, but to a drop for males; adopting the WHO diet would lead to slightly lower levels of emissions relative to the status quo for both genders. Figure 12 in Appendix G further shows modelled shifts in GHGE by income decile.

**FIGURE 7:**
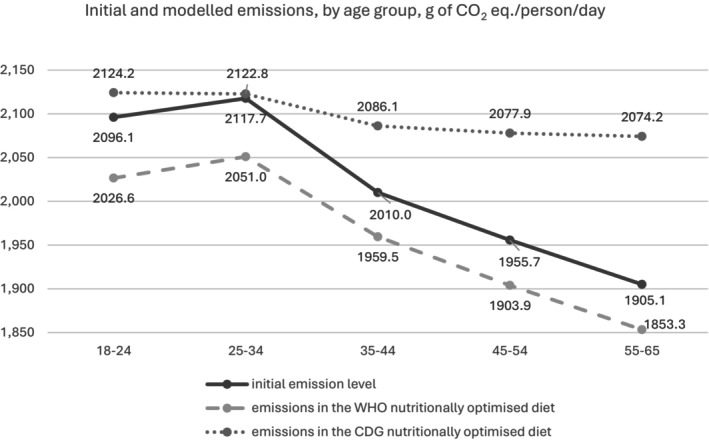
Initial versus modelled emissions, by age group, g of CO_2_ eq./person/day.

Typically, as Figure 7 exhibits, modelled average emissions follow a similar trend to initial emissions when disaggregated by age group. Apart from the CDG scenario where GHGE peak for the 18–24 years age group and gradually decrease thereafter, initial and WHO optimised emissions marginally increase from the first to the second age group and then follow a decreasing pattern. The CDG optimised diet corresponding to the youngest age segment in the dataset has the highest magnitude of emissions. Older consumers generate the highest rise in carbon footprint when their diets adjust according to CDG recommendations.

**FIGURE 8:**
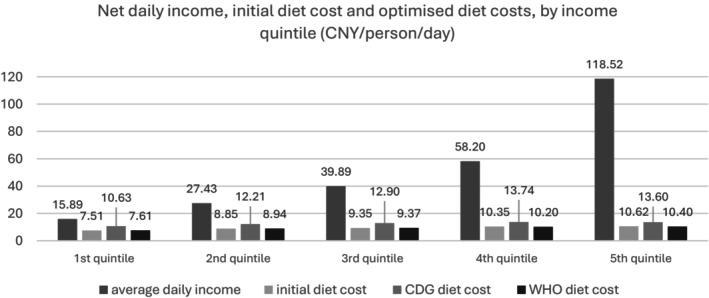
Average net daily income, initial diet cost and optimised diet costs, by income quintile (Chinese Yuan (CNY)/person/day).

Figure 8 shows that although the gap between the mean income of the fourth and fifth income quintiles is wide, the initial food cost varies only slightly. Optimised diet costs are also similar between these two quintiles. For the first and second quintiles, the cost of the CDG nutritionally optimised diet appears to be unjustifiably high, especially since the WHO scenario confirms a nutritional diet can be achieved with a significantly lower expense.

### 3.3 Models With Caps on Greenhouse Gas Emissions

The CDG models imposing GHGE reductions of 10% and 20% only converge for a small number of individuals, while the WHO models find feasible solutions for most individuals in the data set. We focus on a data set of 3260 individuals (36% of the total) with identifiable CDG and WHO diets that reduce emissions by 10% and 20%. Both WHO and CDG modelled diets yield 2357.49 g CO_2_ eq. per person per day under the 10% emissions reduction scenario, and 2095.66 g CO_2_ eq. per person per day under the 20% reduction case. Figures 13 and 14 in Appendix G display the optimised diet compositions by gender. Maintaining diet affordability and achieving emissions reductions are possible for these individuals due to their higher baseline incomes and above‐average diet‐related emissions (of 2619.43 g CO_2_ eq./person/day).

**FIGURE 9:**
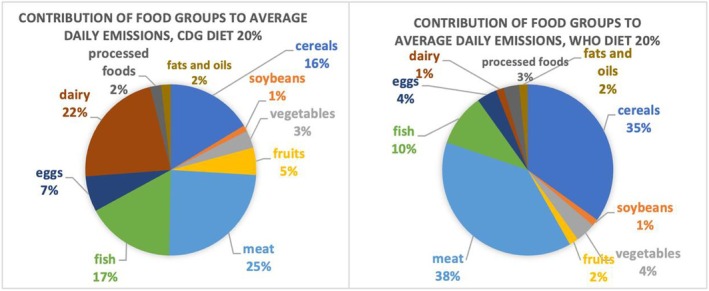
Food contributions to daily diet‐related greenhouse gas emissions (GHGE), Chinese Dietary Guidelines (CDG) and World Health Organization (WHO) optimised diets requiring a 20% reduction in emissions.

Figure 9 shows the mean contribution to daily GHGE from each food group under the CDG and WHO diets with a 20% emissions reduction. In the CDG model, meat, dairy and fish are the largest contributors, while fruits and vegetables account for just 8% of daily CO_2_ output. In the WHO diet, meat, cereals and fish together make up 83% of emissions, with other foods contributing minimally. One of the main differences between the two scenarios is that dairy contributes significantly more to emissions in the CDG diet.

Table 3 shows diet cost shifts by age. CDG diets are more costly than the WHO alternative, and they become gradually more expensive with age, with the largest percentage increase in costs occurring for consumers over 44 years. Diet costs gradually increase across age groups because older individuals have diets that diverge more from the CDG recommendations, and bridging the gap between their consumption levels and recommended ones results in a higher level of spending. WHO diet costs are negatively correlated with emissions, but no clear age‐related pattern is found. However, the 35–44 year age group shows the smallest percentage decrease in food expenditure across both WHO scenarios.

**TABLE 3:** Changes in average diet costs by age group (%) in the optimised Chinese Dietary Guidelines (CDG) and World Health Organisation (WHO) diets imposing a 10% and 20% emissions reduction relative to the baseline diet.

Percentage change in diet cost (relative to status quo)				
	10% emissions reduction diet		20% emissions reduction diet	
	CDG	WHO	CDG	WHO
Average cost	+22%	−5%	+18%	−8%
By age group (years)				
18–24	+20%	−6%	+16%	−10%
25–34	+19%	−6%	+15%	−9%
35–44	+22%	−4%	+18%	−7%
45–54	+24%	−5%	+19%	−8%
55–65	+24%	−6%	+19%	−9%

### 3.4 WHO Scenarios With Higher Levels of Greenhouse Gas Emissions Reduction

While CDG diets are unfeasible for much of the sample, the WHO guidelines allow comparison across a broader range of GHGE reduction targets, so we sought WHO‐optimised diets for greater CO_2_ reductions. Retaining only individuals for whom all WHO models converge (including the nutritionally optimised model and those with additional 10%, 20%, 30% and 40% emissions reductions, averaging a stepwise 200 g CO_2_ eq. reduction per person per day from the baseline of 1970 g CO_2_ eq./day) results in a data set of 8473 individuals.

**FIGURE 10:**
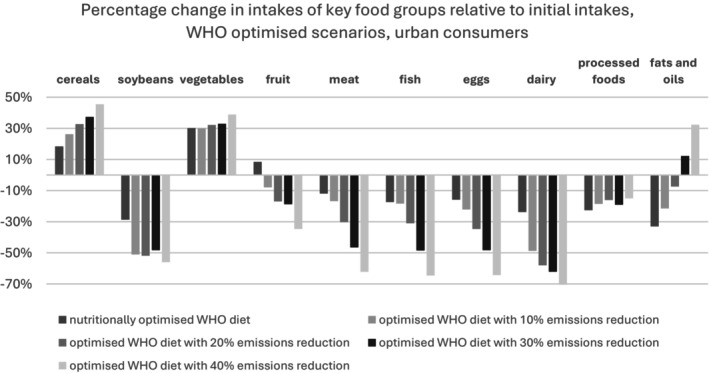
World Health Organization (WHO) optimised scenarios, urban consumers.

**FIGURE 11:**
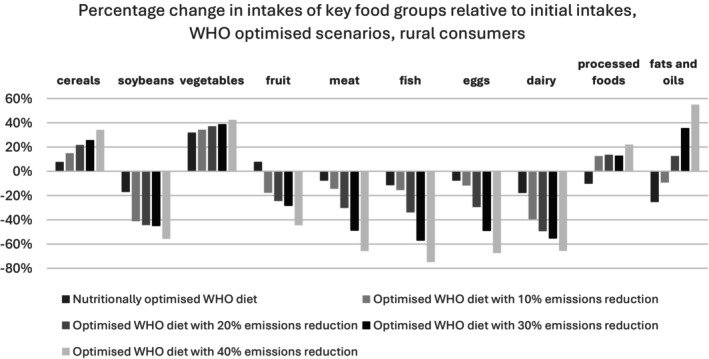
World Health Organization (WHO) optimised scenarios, rural consumers.

Figures 10 and 11 show that cereal and vegetable intakes rise across all optimised WHO scenarios for both urban and rural consumers. Soybeans, meat, fish, eggs and dairy intakes decrease, especially in models with high GHGE reductions. Fruit consumption increases in the nutritionally optimised diet but declines in other models. Urban consumers in all scenarios eat less processed food relative to the baseline, whereas rural consumers (except in the nutritionally optimised case) see an increase in processed food consumption. Diets with high CO_2_ reductions also lead to higher fat and oil intakes, regardless of residence.

Additional modes with higher levels of GHGE reduction were run, but we concluded the WHO diet stops being varied and, most likely, acceptable to the public above a 30% decrease in emissions—our finding is in line with previous work (Green et al. 2015). Beyond this point, there are clear nutritional and environmental trade‐offs, with diets being completely vegetarian and consisting of mainly cereals and vegetables. CDG diets were not discussed at this level, as the CDG model with a 30% emissions reduction provides solutions for only 28% of the initial data set. This is due to the diets becoming unaffordable, as many individuals initially under‐consume compared to the CDG recommendations.

## 4 Discussion

A comparison of the optimised WHO and CDG diets without environmental constraints reveals they differ significantly in terms of the individual foods they recommend. While we cannot determine which is more nutritionally suitable, we argue that adopting the WHO diet would require less change from observed diets. The discrepancies in food group intakes between the CDG and WHO models likely reflect underlying inconsistencies in the nutrient profiles of diets that conform to each set of guidelines. Additionally, our modelling focuses on macronutrient analysis, and we acknowledge that the exclusion of micronutrient considerations, including fatty acid profiles (saturated and unsaturated fats), is likely to influence the results.

The WHO nutritionally optimised diet costs only slightly more than the observed diet, making it affordable and acceptable to a broad audience. In contrast, the CDG model significantly increases diet costs (about 35% for men and 44% for women), making it economically unfeasible for many, especially lower‐income households who already struggle to afford proper diets, and for whom diet costs are expected to rise the most. CDG diets are too expensive for a large portion of the population, primarily due to higher recommended dairy consumption.

China's growing health awareness has driven increased demand for milk due to its perceived benefits, particularly as a source of calcium and protein to address nutritional deficiencies (Daxue Consulting 2022). While lactose intolerance poses challenges—92% of adults are lactose‐intolerant—the market has adapted by promoting products low in lactose such as yoghurt (Daxue Consulting 2022). However, given the low baseline dairy intake, promoting dairy conflicts with sustainability principles due to the high GHGE associated with dairy production. The EAT‐Lancet Commission (Willett et al. 2019) advises moderate dairy intake (0 − 500 g/day) as optional in health‐promoting diets and emphasises adapting dietary guidelines to suit regional and cultural contexts. Given that milk has not traditionally been part of Chinese diets, implementing these recommendations in China likely involves continuing to promote lactose‐free or fermented dairy products to balance nutritional goals with cultural and environmental considerations.

Adopting WHO recommendations would reduce daily emissions by 2.8% compared to observed levels. In contrast, the CDG diet would increase the carbon footprint by 5.1% on average, with a 4% decrease for males and a 15% increase for females. In the CDG scenario, reduced meat and cereal intake is outweighed by higher emissions from other foods, leading to an overall increase in GHGE. The WHO diet, however, reduces all animal product intakes, resulting in emissions savings that offset the rise in emissions from other foods, achieving environmental benefits. We argue that reducing meat consumption supports both environmental sustainability and affordability while increasing fruit and vegetable intakes balances environmental costs with nutritional benefits and dietary diversity. We believe recommending more dairy and fish is less justified due to their significant ecological impact. However, our study focuses on macronutrient analysis, and given the nutrient density of animal proteins—particularly their role in providing essential micronutrients like iron, zinc and B12—this constitutes a limitation in our analysis.

China faces a major demographic shift, with the 50+ years population set to grow by 250 million by 2050, as the under‐50 years population declines by a similar number (Eberstadt 2019). We showed that older adults have lower‐emission diets, whereas younger generations (18–24 years) consume more animal protein, leading to higher‐emission diets likely to persist with age. Since younger individuals in 2011 already had diets high in emissions, it is likely that, now aged 32–38 years, this cohort continues to contribute to elevated emissions. Hence, the demographic transition presents a critical challenge of rising GHGE, driven by younger individuals' higher‐emission diets, making early dietary interventions essential for fostering sustainable habits. We also found that diet costs increase with age, indicating that individuals may need to allocate a larger share of their income to align with CDG recommendations that include higher levels of animal proteins. From a systems perspective, meeting CDG targets will require a significant expansion in domestic and imported animal protein production, exacerbating China's growing reliance on food imports (Liu 2023), and adding more pressure on global food supply chains and environmental resources. This is incoherent with China's aforementioned net‐zero commitments.

Our results indicate that optimising diets could reduce the intake of certain key food groups, potentially leading to deficiencies in essential micronutrients. Given the importance of a balanced micronutrient profile for health, future research should address how dietary optimisation strategies can be balanced with the need for sufficient micronutrient consumption, to ensure that health outcomes are not compromised. In the scenarios with a more than 30% reduction in CO_2_ eq., there is a considerable trade‐off between nutrition and environmental outcomes. The concern extends beyond a reduction in dietary variety to the potential inadequacy of micronutrient intake, which likely fails to meet recommended levels. Several studies modelling more sustainable dietary patterns have similarly concluded that such diets might not be healthy and nutritionally adequate (Green et al. 2015), (Davies et al. 2023).

### 4.1 Strengths and Limitations

This study is the first, to our knowledge, to use China data to model GHGE shifts resulting from adopting CDG and WHO guidelines while ensuring diet affordability. It highlights trade‐offs between nutritional, environmental and economic goals and estimates the maximum carbon abatement achievable with affordable, acceptable diets for Chinese consumers.

A key limitation of this study, like similar research on food consumption and GHGE, is its reliance on approximations due to limited data. For example, protein quality and micronutrient bioavailability, while important, especially when considering consumption of animal sources versus plant‐based foods, were not included in the model due to data availability constraints. Given the CDG's goal to address nutrient deficiencies (China Nutrition Society 2016), the lack of micronutrient analysis is a shortcoming. Nevertheless, the findings provide valuable insights into macronutrient consumption and its impacts.

Our work relies on total sugars in processed foods as a proxy for added sugars. On one hand, this may overestimate added sugars, as it includes naturally occurring sugars in products like fruit and vegetable juices. On the other hand, some foods containing added sugars may fall outside the considered categories, potentially underestimating sugar intakes. Given the data limitations, we believe that this is the best approach to provide meaningful insights into sugar consumption.

Moreover, we use 2011 food consumption data, but China's rapid nutrition transition likely means significant dietary changes have occurred since, and our analysis does not capture those changes. With fast urbanisation and lifestyle shifts, the potential for policy‐driven improvements in diets may now be greater (He et al. 2019). Moreover, self‐reported dietary data likely underestimates total calories, GHGE, and unhealthy food intake 2015, leading to conservative findings on the unhealthiness of the Chinese diet and its environmental footprint (Green et al. ). Although China has historically pushed for self‐sufficiency in food production, over the past couple of decades, its reliance on imported food has grown significantly, making it the largest importer of products such as soybeans, corn, wheat, rice and dairy. From 2000 to 2020, the country's food self‐sufficiency ratio declined from 93.6% to 65.8% (Liu 2023). Therefore, if our analysis was replicated with 2024 data, we would need to more carefully consider differences in emissions between imported and domestically produced foods.

## 5 Policy Implications

This paper contributes to the debate on whether enhancing dietary quality in China and emerging economies in general can result in environmental co‐benefits. This is particularly relevant since the bulk of the research to date has focused on high‐income countries and, to our knowledge, no previous studies on China have attempted to use an optimisation model to compare the nutritional, environmental and economic impacts of the recommendations contained in the CDG and WHO guidelines. Our paper's comprehensive approach provides valuable implications for policymakers and nutritionists, highlighting the need for holistic strategies that balance nutrition, cost and environmental considerations.

Our analysis shows that adopting the CDG would increase environmental burdens, while following WHO recommendations would slightly lower food‐related emissions, with the potential for further reductions without significant dietary changes. This contrast arises mainly because, unlike the CDG, the WHO guidelines do not mandate minimum fish and dairy intakes—nutrient‐rich but high‐carbon‐footprint foods underconsumed in baseline Chinese diets. Increasing consumption of these two food groups could amplify the dietary environmental burden unless offset by reduced intakes of other foods. While our analysis uses the 2016 CDG, the 2022 guidelines suggest similar meat and fish levels but higher dairy intakes, likely leading to even greater emissions in modelled diets. Thus, an essential implication of our paper is the development of dietary guidelines that not only promote nutritional balance but also consider the environmental impact of food choices.

Our findings highlight the need for policymakers to consider the heterogeneity of diets across socio‐economic groups when designing policies targeting the nutrition‐environment link. Given the disparities in lifestyles, food preferences and behavioural responses among these groups, policy interventions such as healthy eating campaigns and food taxes/subsidies must be tailored to address these differences, as they can otherwise exacerbate inequalities in nutritional and environmental outcomes. For example, we find that over‐consumption and the adoption of Western‐style diets are more common among younger, high‐income, urban, male Chinese consumers. Policymakers have an opportunity to improve dietary quality within this group, while simultaneously delivering environmental benefits.

Relying on WHO results, we argue that the maximum diet‐related GHGE reduction policymakers could realistically aim for is 30%, as further reductions might require switching to diets that are significantly less diverse, potentially reducing their acceptability among the public. This finding aligns with previous research (Green et al. 2015). Given the composition of the computed low‐emission diets, achieving effective decarbonisation necessitates substantial changes in dietary patterns, with resulting diets potentially lacking in health‐supporting micronutrients. This highlights the need for nutritionists and policymakers to exercise caution when revising the recommendations guiding this nutrition transition. Dietary guidelines must be nutritionally balanced and environmentally considerate, yet also flexible enough to accommodate regional and socioeconomic differences, ensuring they are both practical and accessible for diverse populations. Since the WHO model yields a solution for virtually all individuals in the data set, diets meeting these guidelines could be achieved even for the most disadvantaged segments of the population. Future research could examine integrating these guidelines into local policy frameworks, focusing on their effectiveness across regions, socioeconomic groups and food systems.

## Ethics Statement

This study was approved by the Research Ethics Committee at SOAS, University of London (100_SC_REP_OCT18), due to its use of community‐level data from the *China Health and Nutrition Survey (CHNS)*.

## Conflicts of Interest

The authors declare no conflicts of interest.

## Supporting information


Data S1.

## Data Availability

The food consumption and socio‐economic data that support the findings of this study are openly available from the *China Health and Nutrition Survey* (*CHNS*) at https://www.cpc.unc.edu/projects/china. The community price data can be requested from the *CHNS* at https://www.cpc.unc.edu/projects/china/data/linkages. Restrictions apply to the availability of these data, which were used under a data use agreement.
