# Supplementary material for: Consumption and Greenhouse Gas Emissions Impacts of Population‐Wide Adoption of Dietary Guidelines in China
Source: Nutr Bull. 2025 Apr 18;50(2):326–39. doi: 10.1111/nbu.70008 (PMC12147054; doi:10.1111/nbu.70008)
Supplement: Supplementary file 1 — Data S1. [file NBU-50-326-s001.docx]

# Supplementary information

**Appendix A – Price elasticities and budget shares**

| **Group**  **#** | **Food group** | **Own-price elasticity and corresponding elasticity**  **category** | **Budget share (in 2011)** |
| --- | --- | --- | --- |
| 1 | Wheat | Wheat (-0.945) | 0.023 |
| 2 | Rice | Rice (-0.598) | 0.042 |
| 3 | Corn | Coarse grains (-0.859) | 0.001 |
| 4 | Coarse grains, beans and tubers | Coarse grains (-0.859) | 0.016 |
| 5 | Soybeans, nuts and seeds | Coarse grains (-0.859) | 0.011 |
| 6 | Vegetables and mushrooms | Vegetables (-0.568) | 0.015 |
| 7 | Leafy greens | Vegetables (-0.568) | 0.020 |
| 8 | Fruit | Fruit (-0.708) | 0.021 |
| 9 | Pork | Pork (-0.738) | 0.061 |
| 10 | Beef | Beef (-0.607) | 0.010 |
| 11 | Mutton and other red meat | Mutton (-1.055) | 0.004 |
| 12 | Poultry and other white meat | Poultry (-0.731) | 0.012 |
| 13 | Dairy | Dairy products (-0.807) | 0.008 |
| 14 | Eggs | Eggs (-0.626) | 0.010 |
| 15 | Fish | Aquatic products (-0.527) | 0.013 |
| 16 | Non-alcoholic beverages: sugary  drinks, fruit juice, tea and coffee | Soft drinks (-0.92)* | 0.002 |
| 17 | Processed foods | Soft drinks (-0.92)* | 0.007 |
| 18 | Sweets | Sugar (-0.493) | 0.001 |
| 19 | Fats and oils | Edible oils (-0.481) | 0.011 |
| Data sources: the *soft drinks* elasticity was taken from (Seale 2012), while all other values were obtained from (Chen et al. 2016); Budget shares represent the authors’ calculations based on CHNS 2011 consumption and price data | | | |

***Table 1:*** *Elasticities and budget shares*

**References:**

Chen, Danhong, David Abler, De Zhou, Xiaohua Yu, and Wyatt Thompson. 2016. “A Meta-Analysis of Food Demand Elasticities for China.” *Applied Economic Perspectives & Policy* 38 (1): 50–72. https://doi.org/10.1093/aepp/ppv006.

Seale, James. 2012. “Food Consumption Trends in Urban China: Opportunities and Implications for Mekong Region Agriculture,” 45.

**Appendix B - Calculation of GHGE**

In many instances, we retrieved from the literature a wide range of emission estimates for the same food item, and we organised these data using the approach in Vieux et al. (2012) who account for the variability in the GHGE data by assuming these follow a log-normal probability distribution (since all emission data are positive and usually skewed to the right). For each food category, they obtain a 95% confidence interval for emissions by using the (geometric) mean of the assumed distribution and its (geometric) standard deviation. Following the above procedure, when different values for the same food were found in the literature, we also assumed a log-normal distribution of these values, and we calculated their geometric mean (since the mean of a log-normal distribution is the geometric mean). This mean then represented the carbon footprint value attributed to that particular food item in all subsequent calculations. For instance, our searches revealed 11 different values for the farm gate GHGE associated with *rice.* Therefore, we computed the geometric mean of all these values, in order to determine the emissions from *rice.* We tackled in a similar way many other food items, such as *wheat* (for which we uncovered 9 different values estimated in the literature)*, pork* (six different values), *cucumber* (three values), etc.

We acknowledge the variation in emissions within each food group as influenced by factors like growing conditions, agricultural practices, and regional differences, can be significant. While the analysis aggregates emissions at the food group level to simplify the approach, we recognise that these variations can affect the accuracy and confidence of estimates when applied to individual foods. Future research is needed to explore more granular data to capture these differences more effectively.

Furthermore, the carbon footprints for several types of dairy excluding milk were calculated by using the emissions figure for *milk* and then multiplying that number with the extraction rates as given in the FAO conversion table (FAO, n.d.): butter made from cow milk was assumed to have an extraction rate of 4%, whole milk condensed 38%, cheese (made from whole cow milk) 15% and evaporated milk (assumed to be the same as powder milk) 33%. This approach is inspired by the work of Green et al. (2015) who employ FAO conversion factors when computing carbon footprints for dairy products.

The carbon footprint corresponding to the processing and packaging stage was obtained from Green et al. (2018). Emissions resulting from storing these products at retail points as well as cooking-related emissions were retrieved from Green et al. (2018) and Röös et al. (2015). All foods were assumed to be stored before being sold. Extrapolations from similar foods were performed when necessary. In Venkat (2011), 50% of the consumer-accounted food waste is seen as taking place pre-cooking and the other half post-cooking. The same assumption is maintained in the present analysis. Food losses and food waste percentages for each food category and at different stages of the life cycle were determined using data on Industrialised Asia, available in the 2011 FAO report on food losses and food waste (Gustavsson et al. 2011). Since China-specific emission estimates from food transportation were not found, we calculated these and found them to be equal to 0.03 kgs of CO_2_ eq. per kg of food transported.

Following Pathak et al. (2010), emissions from transportation were computed based on the assumption that food items in China are mainly transported by truck. Information on fuel intensity of rigid trucks in China was retrieved from Delgado et al. (n.d.). The authors assume the China cycle for rigid trucks consists of three segments: urban, rural and motorway, included with weights of 10%, 60% and 30%, respectively. The average speed for rigid trucks is 51.3 km/h and their maximum payload is 6.2 tonnes. Since there is a common trend of overloading trucks in China, the representative payload in the authors’ simulation is almost 100% of its original value. A cycle consists of the truck departing fully (or almost fully) loaded and returning empty. The authors calculate the average fuel consumption per 100 km for a rigid truck with an average payload of 3045 kg to be 21.2 litres, by assuming the truck departs close to full capacity (6090 kgs) and returns empty.

We utilised their findings and calculated the carbon footprint of fuel by multiplying the amount of fuel consumed (in litres) by the emission factor of fuel. Cao et al. (2014) find an emission factor of 2.56 Kg of CO_2_ equivalent per litre of diesel oil used. Their results are in line with the 2.6 Kg of CO_2_ eq. per litre from Pathak et al. (2010). Based on information on the average food miles inside China that we retrieved from Xia et al. (2016), we assumed that food traverses 175 Km before it reaches supermarket shelves. Using the figures listed here, we computed the value of emissions from transportation to be 0.03 Kgs of CO_2_ eq. per kg of food transported.

**Why using domestic GHGE data is suitable**

Although China has historically pushed for self-sufficiency in food production, over the past few years its food imports have increased considerably. Regarding cereals, domestic production matches consumption, but meat and vegetable imports have risen tremendously (Chinapower 2017). We computed the proportion of imports in the total domestic food supply for 23 major food groups consumed in China, in 2011 and concluded that the majority of the food consumed in China in 2011 was produced inside the country. For instance, 460,367 Kilotonnes of cereals were available for internal consumption in 2011, out of which a mere 2.75% were imported. At the same time, the domestic supply of vegetables was 552,268 Kilotonnes, with imports representing only 0.31%.

Only one of the food groups – soybeans – was more than 50% imported, but since the proportion of this group in the overall diet is not that significant, we argue employing China-specific production data is suitable. Our approach is in agreement with the work of Green et al. (2018) who estimate the level of GHGE from Indian diets and find two major food groups for which imports constitute more than half of the domestic supply. They also argue that using emission estimates from India is appropriate since the two food groups only represent a small percentage in consumption.

**Computing the level of emissions associated with each food group**

First, we calculated the percentage, by weight, in the total group consumption of the foods for which we had GHGE estimates. We did this by aggregating across all individuals in the 2011 survey wave. These percentages then provided the weights we used when computing the weighted average of emissions for each food group.

As an example, for *fruit*, we had the emissions for eight separate types of fruit: *apple, orange, tangerine, other citrus, banana, peach, pear* and *grapes.* Thus, we first determined, for each of these fruits, their proportion in the overall consumption of the *fruit* group. Next, we used these proportions together with the emission level of each individual food item, to calculate the weighted emissions average for the *fruit* category.

**References**

Cao, L., Li, M., Wang, X., Zhao, Z., Pan, X., 2014. n.d. “Life Cycle Assessment of Carbon Footprint for Rice Production in Shanghai. Acta Ecol. Sin. 491e499 (In Chinese).”

Chen, Danhong. Abler David, De Zhou, Xiaohua Yu, and Wyatt Thompson. 2016. “A Meta-Analysis of Food Demand Elasticities for China.” *Applied Economic Perspectives & Policy* 38 (1): 50–72. <https://doi.org/10.1093/aepp/ppv006>.

Chinapower. 2017. “How Is China Feeding Its Population of 1.4 Billion?” ChinaPower Project (blog). January 25, 2017. <https://chinapower.csis.org/china-food-security/>. Accessed 2018-05-13.

Delgado, O., R. Muncrief, J. Miller, and B. Sharpe. n.d. “ESTIMATING THE FUEL EFFICIENCY TECHNOLOGY POTENTIAL OF HEAVY-DUTY TRUCKS IN MAJOR MARKETS AROUND THE WORLD.” International Council on Clean Transportation (ICCT).

FAO. n.d. “Technical Conversion Factors for Agricultural Commodities.”

Green, Rosemary F., Edward J. M. Joy, Francesca Harris, Sutapa Agrawal, Lukasz Aleksandrowicz, Jon Hillier, Jennie I. Macdiarmid, et al. 2018. “Greenhouse Gas Emissions and Water Footprints of Typical Dietary Patterns in India.” *Science of The Total Environment* 643 (December):1411–18. <https://doi.org/10.1016/j.scitotenv.2018.06.258>.

Green, Rosemary, James Milner, Alan D. Dangour, Andy Haines, Zaid Chalabi, Anil Markandya, Joseph Spadaro, and Paul Wilkinson. 2015. “The Potential to Reduce Greenhouse Gas Emissions in the UK through Healthy and Realistic Dietary Change.” *Climatic Change* 129 (1–2): 253–65. https://doi.org/10.1007/s10584-015-1329-y.

Gustavsson, Jenny, Christel Cederberg, and Ulf Sonesson. 2011. *Global Food Losses and Food Waste: Extent, Causes and Prevention; Study Conducted for the International Congress Save Food! At Interpack 2011, [16 - 17 May], Düsseldorf, Germany*. Rome: Food and Agriculture Organization of the United Nations.

Pathak, H., N. Jain, A. Bhatia, J. Patel, and P.K. Aggarwal. 2010. “Carbon Footprints of Indian Food Items.” *Agriculture, Ecosystems & Environment* 139 (1–2): 66–73. <https://doi.org/10.1016/j.agee.2010.07.002>.

Röös, Elin, Hanna Karlsson, Cornelia Witthöft, and Cecilia Sundberg. 2015. “Evaluating the Sustainability of Diets–Combining Environmental and Nutritional Aspects.” *Environmental Science & Policy* 47 (March): 157–66. <https://doi.org/10.1016/j.envsci.2014.12.001>.

Venkat, Kumar. 2011. “The Climate Change and Economic Impacts of Food Waste in the United States.” *International Journal on Food System Dynamics* 2 (4): 431–46.

Vieux, F., N. Darmon, D. Touazi, and L. G. Soler. 2012. “Greenhouse Gas Emissions of Self Selected Individual Diets in France: Changing the Diet Structure or Consuming Less?” *Ecological Economics* 75 (C): 91–101.

Xia, Longlong, Chaopu Ti, Bolun Li, Yongqiu Xia, and Xiaoyuan Yan. 2016. “Greenhouse Gas Emissions and Reactive Nitrogen Releases during the Life-Cycles of Staple Food Production in China and Their Mitigation Potential.” *Science of The Total Environment* 556 (June): 116–25. <https://doi.org/10.1016/j.scitotenv.2016.02.204>.

﻿

| **Production-level emissions and data sources** | | | | | |
| --- | --- | --- | --- | --- | --- |
| **group  #** | **food group** | **foods** | **GHGE: kg of CO_2_ eq.  per kg of product** | **region** | **source** |
| 1 | wheat | wheat | 0.410 | China | Jianyi, Lin, Hu Yuanchao, Cui Shenghui, Kang Jiefeng, and Xu Lilai. 2015. “Carbon Footprints of Food Production in China (1979–2009).” Journal of Cleaner Production 90 (March): 97–103. https://doi.org/10.1016/j.jclepro.2014.11.072. |
| 1 | wheat | wheat | 0.454 | China | Xu, Xiaoming, and Ying Lan. 2016. “A Comparative Study on Carbon Footprints between Plant- and Animal-Based Foods in China.” Journal of Cleaner Production 112 (January): 2581–92. https://doi.org/10.1016/j.jclepro.2015.10.059. |
| 1 | wheat | wheat | 0.750 | China | Zhang, Dan, Jianbo Shen, Fusuo Zhang, Yu’e Li, and Weifeng Zhang. 2017. “Carbon Footprint of Grain Production in China.” Scientific Reports 7 (1): 4126. https://doi.org/10.1038/s41598-017-04182- |
| 1 | wheat | wheat | 0.711 | China | Zhang, G., Wang, X., Zhang, L., Xiong, K., Zheng, C., Lu, F., Zhao, H., Zheng, H., Ouyang, Z., 2018. Carbon and water footprints of major cereal crops production in China. J. Clean. Prod. 194, 613–623. https://doi.org/10.1016/j.jclepro.2018.05.024 |
| 1 | wheat | wheat | 0.510 | China | Chen et al. (2011) cited in Luo, Ting, Qian Yue, Ming Yan, Kun Cheng, and Genxing Pan. 2015. “Carbon Footprint of China’s Livestock System – a Case Study of Farm Survey in Sichuan Province, China.” Journal of Cleaner Production 102 (September): 136–43. https://doi.org/10.1016/j.jclepro.2015.04.077. |
| 1 | wheat | wheat | 0.140 | China | Cheng, K., Yan, M., Nayak, D., Pan, G.X., Smith, P., Zheng, J.F., Zheng, J.W., 2015. Carbon footprint of crop production in China: an analysis of National Statistics data. J. Agric. Sci. 153, 422–431. https://doi.org/10.1017/S0021859614000665 |
| 1 | wheat | wheat | 0.600 | China | Yue, Qian, Xiangrui Xu, Jonathan Hillier, Kun Cheng, and Genxing Pan. 2017. “Mitigating Greenhouse Gas Emissions in Agriculture: From Farm Production to Food Consumption.” Journal of Cleaner Production 149 (April): 1011–19. https://doi.org/10.1016/j.jclepro.2017.02.172. |
| 1 | wheat | wheat | 0.650 | China | Yan, Ming, Kun Cheng, Ting Luo, Yu Yan, Genxing Pan, and Robert M. Rees. 2015. “Carbon Footprint of Grain Crop Production in China – Based on Farm Survey Data.” Journal of Cleaner Production 104 (October): 130–38. https://doi.org/10.1016/j.jclepro.2015.05.058. |
| 1 | wheat | wheat | 0.151 | China | Liu, Weiwei, Guo Zhang, Xiaoke Wang, Fei Lu, and Zhiyun Ouyang. 2018. “Carbon Footprint of Main Crop Production in China: Magnitude, Spatial-Temporal Pattern and Attribution.” Science of The Total Environment 645 (December): 1296–1308. https://doi.org/10.1016/j.scitotenv.2018.07.104. |
| 1 | wheat | wheat flour | 0.575 | China | Xu, Xiaoming, and Ying Lan. 2016. “A Comparative Study on Carbon Footprints between Plant- and Animal-Based Foods in China.” Journal of Cleaner Production 112 (January): 2581–92. https://doi.org/10.1016/j.jclepro.2015.10.059. |
| 1 | wheat | wheat flour | 1.180 | China | Xia, Longlong, Chaopu Ti, Bolun Li, Yongqiu Xia, and Xiaoyuan Yan. 2016. “Greenhouse Gas Emissions and Reactive Nitrogen Releases during the Life-Cycles of Staple Food Production in China and Their Mitigation Potential.” Science of The Total Environment 556 (June): 116–25. https://doi.org/10.1016/j.scitotenv.2016.02.204. |
| 2 | rice | rice | 1.750 | China | Jianyi, Lin, Hu Yuanchao, Cui Shenghui, Kang Jiefeng, and Xu Lilai. 2015. “Carbon Footprints of Food Production in China (1979–2009).” Journal of Cleaner Production 90 (March): 97–103. https://doi.org/10.1016/j.jclepro.2014.11.072. |
| 2 | rice | rice | 0.901 | China | Xu, Xiaoming, and Ying Lan. 2016. “A Comparative Study on Carbon Footprints between Plant- and Animal-Based Foods in China.” Journal of Cleaner Production 112 (January): 2581–92. https://doi.org/10.1016/j.jclepro.2015.10.059. |
| 2 | rice | rice (processed for eating) | 2.490 | China | Xia, Longlong, Chaopu Ti, Bolun Li, Yongqiu Xia, and Xiaoyuan Yan. 2016. “Greenhouse Gas Emissions and Reactive Nitrogen Releases during the Life-Cycles of Staple Food Production in China and Their Mitigation Potential.” Science of The Total Environment 556 (June): 116–25. https://doi.org/10.1016/j.scitotenv.2016.02.204. |
| 2 | rice | rice | 1.600 | China | Zhang, Dan, Jianbo Shen, Fusuo Zhang, Yu’e Li, and Weifeng Zhang. 2017. “Carbon Footprint of Grain Production in China.” Scientific Reports 7 (1): 4126. https://doi.org/10.1038/s41598-017-04182- |
| 2 | rice | rice | 1.225 | China | Yue, Qian, Xiangrui Xu, Jonathan Hillier, Kun Cheng, and Genxing Pan. 2017. “Mitigating Greenhouse Gas Emissions in Agriculture: From Farm Production to Food Consumption.” Journal of Cleaner Production 149 (April): 1011–19. https://doi.org/10.1016/j.jclepro.2017.02.172. |
| 2 | rice | rice | 1.447 | China | Zhang, G., Wang, X., Zhang, L., Xiong, K., Zheng, C., Lu, F., Zhao, H., Zheng, H., Ouyang, Z., 2018. Carbon and water footprints of major cereal crops production in China. J. Clean. Prod. 194, 613–623. https://doi.org/10.1016/j.jclepro.2018.05.024 |
| 2 | rice | paddy rice | 1.360 | China | Chen et al. (2011) cited in Luo, Ting, Qian Yue, Ming Yan, Kun Cheng, and Genxing Pan. 2015. “Carbon Footprint of China’s Livestock System – a Case Study of Farm Survey in Sichuan Province, China.” Journal of Cleaner Production 102 (September): 136–43. https://doi.org/10.1016/j.jclepro.2015.04.077. |
| 2 | rice | rice | 0.800 | China | Yan, Ming, Kun Cheng, Ting Luo, Yu Yan, Genxing Pan, and Robert M. Rees. 2015. “Carbon Footprint of Grain Crop Production in China – Based on Farm Survey Data.” Journal of Cleaner Production 104 (October): 130–38. https://doi.org/10.1016/j.jclepro.2015.05.058. |
| 2 | rice | rice | 1.232 | Shanghai | Cao, L., Li, M., Wang, X., Zhao, Z., Pan, X., 2014, n.d. Life cycle assessment of carbon footprint for rice production in Shanghai. Acta Ecol. Sin. 491e499 (In Chinese). |
| 2 | rice | rice | 0.370 | China | Cheng, K., Yan, M., Nayak, D., Pan, G.X., Smith, P., Zheng, J.F., Zheng, J.W., 2015. Carbon footprint of crop production in China: an analysis of National Statistics data. J. Agric. Sci. 153, 422–431. https://doi.org/10.1017/S0021859614000665 |
| 2 | rice | rice | 0.340 | China | Liu, Weiwei, Guo Zhang, Xiaoke Wang, Fei Lu, and Zhiyun Ouyang. 2018. “Carbon Footprint of Main Crop Production in China: Magnitude, Spatial-Temporal Pattern and Attribution.” Science of The Total Environment 645 (December): 1296–1308. https://doi.org/10.1016/j.scitotenv.2018.07.104. |
| 3 | maize | maize | 0.360 | China | Jianyi, Lin, Hu Yuanchao, Cui Shenghui, Kang Jiefeng, and Xu Lilai. 2015. “Carbon Footprints of Food Production in China (1979–2009).” Journal of Cleaner Production 90 (March): 97–103. https://doi.org/10.1016/j.jclepro.2014.11.072. |
| 3 | maize | maize | 0.321 | China | Xu, Xiaoming, and Ying Lan. 2016. “A Comparative Study on Carbon Footprints between Plant- and Animal-Based Foods in China.” Journal of Cleaner Production 112 (January): 2581–92. https://doi.org/10.1016/j.jclepro.2015.10.059. |
| 3 | maize | maize | 0.480 | China | Zhang, Dan, Jianbo Shen, Fusuo Zhang, Yu’e Li, and Weifeng Zhang. 2017. “Carbon Footprint of Grain Production in China.” Scientific Reports 7 (1): 4126. https://doi.org/10.1038/s41598-017-04182- |
| 3 | maize | maize | 0.120 | China | Cheng, K., Yan, M., Nayak, D., Pan, G.X., Smith, P., Zheng, J.F., Zheng, J.W., 2015. Carbon footprint of crop production in China: an analysis of National Statistics data. J. Agric. Sci. 153, 422–431. https://doi.org/10.1017/S0021859614000665 |
| 3 | maize | maize | 0.330 | China | Yan, Ming, Kun Cheng, Ting Luo, Yu Yan, Genxing Pan, and Robert M. Rees. 2015. “Carbon Footprint of Grain Crop Production in China – Based on Farm Survey Data.” Journal of Cleaner Production 104 (October): 130–38. https://doi.org/10.1016/j.jclepro.2015.05.058. |
| 3 | maize | maize | 0.108 | China | Liu, Weiwei, Guo Zhang, Xiaoke Wang, Fei Lu, and Zhiyun Ouyang. 2018. “Carbon Footprint of Main Crop Production in China: Magnitude, Spatial-Temporal Pattern and Attribution.” Science of The Total Environment 645 (December): 1296–1308. https://doi.org/10.1016/j.scitotenv.2018.07.104. |
| 3 | corn | corn | 0.589 | China | Zhang, G., Wang, X., Zhang, L., Xiong, K., Zheng, C., Lu, F., Zhao, H., Zheng, H., Ouyang, Z., 2018. Carbon and water footprints of major cereal crops production in China. J. Clean. Prod. 194, 613–623. https://doi.org/10.1016/j.jclepro.2018.05.024 |
| 3 | corn | corn | 0.440 | China | Chen et al. (2011) cited in Luo, Ting, Qian Yue, Ming Yan, Kun Cheng, and Genxing Pan. 2015. “Carbon Footprint of China’s Livestock System – a Case Study of Farm Survey in Sichuan Province, China.” Journal of Cleaner Production 102 (September): 136–43. https://doi.org/10.1016/j.jclepro.2015.04.077. |
| 3 | maize | corn | 0.590 | China | Zhongyue, X., Guifang, L., Weijun, X., Yongwu, D., 2018008. Evaluation of greenhouse gas emissions from maize production in China. Chem. Eng. Trans. 1309–1314. https://doi.org/10.3303/CET1870219 |
| 3 | maize | corn | 0.450 | China | Yue, Qian, Xiangrui Xu, Jonathan Hillier, Kun Cheng, and Genxing Pan. 2017. “Mitigating Greenhouse Gas Emissions in Agriculture: From Farm Production to Food Consumption.” Journal of Cleaner Production 149 (April): 1011–19. https://doi.org/10.1016/j.jclepro.2017.02.172. |
| 4 | other cereal | barley | 0.630 | Industrialized Asia: China, Japan, South Korea | Porter, Stephen D., David S. Reay, Peter Higgins, and Elizabeth Bomberg. 2016. “A Half-Century of Production-Phase Greenhouse Gas Emissions from Food Loss & Waste in the Global Food Supply Chain.” Science of The Total Environment 571 (November): 721–29. |
| 4 | other cereal | millet | 0.930 | Industrialized Asia: China, Japan, South Korea | Porter, Stephen D., David S. Reay, Peter Higgins, and Elizabeth Bomberg. 2016. “A Half-Century of Production-Phase Greenhouse Gas Emissions from Food Loss & Waste in the Global Food Supply Chain.” Science of The Total Environment 571 (November): 721–29. |
| 4 | other cereal | oats | 0.930 | Industrialized Asia: China, Japan, South Korea | Porter, Stephen D., David S. Reay, Peter Higgins, and Elizabeth Bomberg. 2016. “A Half-Century of Production-Phase Greenhouse Gas Emissions from Food Loss & Waste in the Global Food Supply Chain.” Science of The Total Environment 571 (November): 721–29. |
| 4 | other cereal | rye | 1.020 | Industrialized Asia: China, Japan, South Korea | Porter, Stephen D., David S. Reay, Peter Higgins, and Elizabeth Bomberg. 2016. “A Half-Century of Production-Phase Greenhouse Gas Emissions from Food Loss & Waste in the Global Food Supply Chain.” Science of The Total Environment 571 (November): 721–29. |
| 4 | other cereal | sorghum | 0.930 | Industrialized Asia: China, Japan, South Korea | Porter, Stephen D., David S. Reay, Peter Higgins, and Elizabeth Bomberg. 2016. “A Half-Century of Production-Phase Greenhouse Gas Emissions from Food Loss & Waste in the Global Food Supply Chain.” Science of The Total Environment 571 (November): 721–29. |
| 4 | other cereal | sorghum | 0.145 | China | Liu, Weiwei, Guo Zhang, Xiaoke Wang, Fei Lu, and Zhiyun Ouyang. 2018. “Carbon Footprint of Main Crop Production in China: Magnitude, Spatial-Temporal Pattern and Attribution.” Science of The Total Environment 645 (December): 1296–1308. https://doi.org/10.1016/j.scitotenv.2018.07.104. |
| 4 | other cereal | other cereals | 0.930 | Industrialized Asia: China, Japan, South Korea | Porter, Stephen D., David S. Reay, Peter Higgins, and Elizabeth Bomberg. 2016. “A Half-Century of Production-Phase Greenhouse Gas Emissions from Food Loss & Waste in the Global Food Supply Chain.” Science of The Total Environment 571 (November): 721–29. |
| 5 | tubers | potato | 0.100 | China | Yue, Qian, Xiangrui Xu, Jonathan Hillier, Kun Cheng, and Genxing Pan. 2017. “Mitigating Greenhouse Gas Emissions in Agriculture: From Farm Production to Food Consumption.” Journal of Cleaner Production 149 (April): 1011–19. https://doi.org/10.1016/j.jclepro.2017.02.172. |
| 5 | tubers | potato | 0.046 | China | Xu, Xiaoming, and Ying Lan. 2016. “A Comparative Study on Carbon Footprints between Plant- and Animal-Based Foods in China.” Journal of Cleaner Production 112 (January): 2581–92. https://doi.org/10.1016/j.jclepro.2015.10.059. |
| 5 | tubers | potato | 0.267 | China | Liu, Weiwei, Guo Zhang, Xiaoke Wang, Fei Lu, and Zhiyun Ouyang. 2018. “Carbon Footprint of Main Crop Production in China: Magnitude, Spatial-Temporal Pattern and Attribution.” Science of The Total Environment 645 (December): 1296–1308. https://doi.org/10.1016/j.scitotenv.2018.07.104. |
| 5 | tubers | roots | 0.190 | China | Jianyi, Lin, Hu Yuanchao, Cui Shenghui, Kang Jiefeng, and Xu Lilai. 2015. “Carbon Footprints of Food Production in China (1979–2009).” Journal of Cleaner Production 90 (March): 97–103. https://doi.org/10.1016/j.jclepro.2014.11.072. |
| 5 | tubers | starchy roots | 0.190 | Industrialized Asia: China, Japan, South Korea | Porter, Stephen D., David S. Reay, Peter Higgins, and Elizabeth Bomberg. 2016. “A Half-Century of Production-Phase Greenhouse Gas Emissions from Food Loss & Waste in the Global Food Supply Chain.” Science of The Total Environment 571 (November): 721–29. https://doi.org/10.1016/j.scitotenv.2016.07.041. |
| 6 | soybean | soybean | 0.470 | China | Xu, Xiaoming, and Ying Lan. 2016. “A Comparative Study on Carbon Footprints between Plant- and Animal-Based Foods in China.” Journal of Cleaner Production 112 (January): 2581–92. https://doi.org/10.1016/j.jclepro.2015.10.059. |
| 6 | soybeans | soybean | 0.100 | China | Cheng, K., Yan, M., Nayak, D., Pan, G.X., Smith, P., Zheng, J.F., Zheng, J.W., 2015. Carbon footprint of crop production in China: an analysis of National Statistics data. J. Agric. Sci. 153, 422–431. https://doi.org/10.1017/S0021859614000665 |
| 6 | soybean | soybean | 0.460 | China | Yue, Qian, Xiangrui Xu, Jonathan Hillier, Kun Cheng, and Genxing Pan. 2017. “Mitigating Greenhouse Gas Emissions in Agriculture: From Farm Production to Food Consumption.” Journal of Cleaner Production 149 (April): 1011–19. https://doi.org/10.1016/j.jclepro.2017.02.172. |
| 7 | beans and pulses | pulses | 0.330 | China | Jianyi, Lin, Hu Yuanchao, Cui Shenghui, Kang Jiefeng, and Xu Lilai. 2015. “Carbon Footprints of Food Production in China (1979–2009).” Journal of Cleaner Production 90 (March): 97–103. https://doi.org/10.1016/j.jclepro.2014.11.072. |
| 7 | beans and pulses | pulses | 0.370 | Industrialized Asia: China, Japan, South Korea | Porter, Stephen D., David S. Reay, Peter Higgins, and Elizabeth Bomberg. 2016. “A Half-Century of Production-Phase Greenhouse Gas Emissions from Food Loss & Waste in the Global Food Supply Chain.” Science of The Total Environment 571 (November): 721–29. https://doi.org/10.1016/j.scitotenv.2016.07.041. |
| 7 | beans and pulses | kidney beans | 0.190 | China | Yue, Qian, Xiangrui Xu, Jonathan Hillier, Kun Cheng, and Genxing Pan. 2017. “Mitigating Greenhouse Gas Emissions in Agriculture: From Farm Production to Food Consumption.” Journal of Cleaner Production 149 (April): 1011–19. https://doi.org/10.1016/j.jclepro.2017.02.172. |
| 8 | vegetables | tomato | 0.028 | China | Xu, Xiaoming, and Ying Lan. 2016. “A Comparative Study on Carbon Footprints between Plant- and Animal-Based Foods in China.” Journal of Cleaner Production 112 (January): 2581–92. https://doi.org/10.1016/j.jclepro.2015.10.059. |
| 8 | vegetables | tomato | 0.170 | Nanjing | Chen et al. (2011) cited in Luo, Ting, Qian Yue, Ming Yan, Kun Cheng, and Genxing Pan. 2015. “Carbon Footprint of China’s Livestock System – a Case Study of Farm Survey in Sichuan Province, China.” Journal of Cleaner Production 102 (September): 136–43. https://doi.org/10.1016/j.jclepro.2015.04.077. |
| 8 | vegetables | tomato | 0.150 | China | Yue, Qian, Xiangrui Xu, Jonathan Hillier, Kun Cheng, and Genxing Pan. 2017. “Mitigating Greenhouse Gas Emissions in Agriculture: From Farm Production to Food Consumption.” Journal of Cleaner Production 149 (April): 1011–19. https://doi.org/10.1016/j.jclepro.2017.02.172. |
| 8 | vegetables | radish | 0.014 | China | Xu, Xiaoming, and Ying Lan. 2016. “A Comparative Study on Carbon Footprints between Plant- and Animal-Based Foods in China.” Journal of Cleaner Production 112 (January): 2581–92. https://doi.org/10.1016/j.jclepro.2015.10.059. |
| 8 | vegetables | radish | 0.060 | China | Yue, Qian, Xiangrui Xu, Jonathan Hillier, Kun Cheng, and Genxing Pan. 2017. “Mitigating Greenhouse Gas Emissions in Agriculture: From Farm Production to Food Consumption.” Journal of Cleaner Production 149 (April): 1011–19. https://doi.org/10.1016/j.jclepro.2017.02.172. |
| 8 | vegetables | cucumber | 0.031 | China | Xu, Xiaoming, and Ying Lan. 2016. “A Comparative Study on Carbon Footprints between Plant- and Animal-Based Foods in China.” Journal of Cleaner Production 112 (January): 2581–92. https://doi.org/10.1016/j.jclepro.2015.10.059. |
| 8 | vegetables | cucumber | 0.100 | Nanjing | Chen et al. (2011) cited in Luo, Ting, Qian Yue, Ming Yan, Kun Cheng, and Genxing Pan. 2015. “Carbon Footprint of China’s Livestock System – a Case Study of Farm Survey in Sichuan Province, China.” Journal of Cleaner Production 102 (September): 136–43. https://doi.org/10.1016/j.jclepro.2015.04.077. |
| 8 | vegetables | cucumber | 0.165 | China | Yue, Qian, Xiangrui Xu, Jonathan Hillier, Kun Cheng, and Genxing Pan. 2017. “Mitigating Greenhouse Gas Emissions in Agriculture: From Farm Production to Food Consumption.” Journal of Cleaner Production 149 (April): 1011–19. https://doi.org/10.1016/j.jclepro.2017.02.172. |
| 8 | vegetables | eggplant | 0.047 | China | Xu, Xiaoming, and Ying Lan. 2016. “A Comparative Study on Carbon Footprints between Plant- and Animal-Based Foods in China.” Journal of Cleaner Production 112 (January): 2581–92. https://doi.org/10.1016/j.jclepro.2015.10.059. |
| 8 | vegetables | eggplant | 0.215 | China | Yue, Qian, Xiangrui Xu, Jonathan Hillier, Kun Cheng, and Genxing Pan. 2017. “Mitigating Greenhouse Gas Emissions in Agriculture: From Farm Production to Food Consumption.” Journal of Cleaner Production 149 (April): 1011–19. https://doi.org/10.1016/j.jclepro.2017.02.172. |
| 8 | vegetables | bell pepper | 0.048 | China | Xu, Xiaoming, and Ying Lan. 2016. “A Comparative Study on Carbon Footprints between Plant- and Animal-Based Foods in China.” Journal of Cleaner Production 112 (January): 2581–92. https://doi.org/10.1016/j.jclepro.2015.10.059. |
| 8 | vegetables | green pepper | 0.225 | China | Yue, Qian, Xiangrui Xu, Jonathan Hillier, Kun Cheng, and Genxing Pan. 2017. “Mitigating Greenhouse Gas Emissions in Agriculture: From Farm Production to Food Consumption.” Journal of Cleaner Production 149 (April): 1011–19. https://doi.org/10.1016/j.jclepro.2017.02.172. |
| 8 | vegetables | cauliflower | 0.125 | China | Xu, Xiaoming, and Ying Lan. 2016. “A Comparative Study on Carbon Footprints between Plant- and Animal-Based Foods in China.” Journal of Cleaner Production 112 (January): 2581–92. https://doi.org/10.1016/j.jclepro.2015.10.059. |
| 8 | vegetables | cauliflower | 0.140 | China | Yue, Qian, Xiangrui Xu, Jonathan Hillier, Kun Cheng, and Genxing Pan. 2017. “Mitigating Greenhouse Gas Emissions in Agriculture: From Farm Production to Food Consumption.” Journal of Cleaner Production 149 (April): 1011–19. https://doi.org/10.1016/j.jclepro.2017.02.172. |
| 8 | vegetables | green beans | 0.060 | China | Xu, Xiaoming, and Ying Lan. 2016. “A Comparative Study on Carbon Footprints between Plant- and Animal-Based Foods in China.” Journal of Cleaner Production 112 (January): 2581–92. https://doi.org/10.1016/j.jclepro.2015.10.059. |
| 8 | vegetables | vegetable average | 0.049 | China | Xu, Xiaoming, and Ying Lan. 2016. “A Comparative Study on Carbon Footprints between Plant- and Animal-Based Foods in China.” Journal of Cleaner Production 112 (January): 2581–92. https://doi.org/10.1016/j.jclepro.2015.10.059. |
| 8 | vegetables | vegetable average | 0.150 | China | Yue, Qian, Xiangrui Xu, Jonathan Hillier, Kun Cheng, and Genxing Pan. 2017. “Mitigating Greenhouse Gas Emissions in Agriculture: From Farm Production to Food Consumption.” Journal of Cleaner Production 149 (April): 1011–19. https://doi.org/10.1016/j.jclepro.2017.02.172. |
| 8 | vegetables | vegetable average | 0.260 | China | Jianyi, Lin, Hu Yuanchao, Cui Shenghui, Kang Jiefeng, and Xu Lilai. 2015. “Carbon Footprints of Food Production in China (1979–2009).” Journal of Cleaner Production 90 (March): 97–103. https://doi.org/10.1016/j.jclepro.2014.11.072. |
| 9 | leafy greens | Chinese cabbage | 0.048 | China | Xu, Xiaoming, and Ying Lan. 2016. “A Comparative Study on Carbon Footprints between Plant- and Animal-Based Foods in China.” Journal of Cleaner Production 112 (January): 2581–92. https://doi.org/10.1016/j.jclepro.2015.10.059. |
| 9 | leafy greens | Chinese cabbage | 0.070 | China | Yue, Qian, Xiangrui Xu, Jonathan Hillier, Kun Cheng, and Genxing Pan. 2017. “Mitigating Greenhouse Gas Emissions in Agriculture: From Farm Production to Food Consumption.” Journal of Cleaner Production 149 (April): 1011–19. https://doi.org/10.1016/j.jclepro.2017.02.172. |
| 9 | leafy greens | cabbage | 0.080 | China | Yue, Qian, Xiangrui Xu, Jonathan Hillier, Kun Cheng, and Genxing Pan. 2017. “Mitigating Greenhouse Gas Emissions in Agriculture: From Farm Production to Food Consumption.” Journal of Cleaner Production 149 (April): 1011–19. https://doi.org/10.1016/j.jclepro.2017.02.172. |
| 9 | leafy greens | cabbage | 0.330 | Nanjing | Chen et al. (2011) cited in Luo, Ting, Qian Yue, Ming Yan, Kun Cheng, and Genxing Pan. 2015. “Carbon Footprint of China’s Livestock System – a Case Study of Farm Survey in Sichuan Province, China.” Journal of Cleaner Production 102 (September): 136–43. https://doi.org/10.1016/j.jclepro.2015.04.077. |
| 9 | leafy greens | cabbage | 0.070 | China | Xu, Xiaoming, and Ying Lan. 2016. “A Comparative Study on Carbon Footprints between Plant- and Animal-Based Foods in China.” Journal of Cleaner Production 112 (January): 2581–92. https://doi.org/10.1016/j.jclepro.2015.10.059. |
| 10 | mushrooms | mushrooms | 0.730 | US | Heller, Martin C., and Gregory A. Keoleian. 2015. “Greenhouse Gas Emission Estimates of U.S. Dietary Choices and Food Loss.” Journal of Industrial Ecology 19 (3): 391–401. https://doi.org/10.1111/jiec.12174. |
| 11 | fruits | apple | 0.167 | China | Xu, Xiaoming, and Ying Lan. 2016. “A Comparative Study on Carbon Footprints between Plant- and Animal-Based Foods in China.” Journal of Cleaner Production 112 (January): 2581–92. https://doi.org/10.1016/j.jclepro.2015.10.059. |
| 11 | fruits | apple | 0.240 | China | “Farm and Product Carbon Footprints of China’s Fruit Production—Life Cycle Inventory of Representative Orchards of Five Major Fruits \| SpringerLink.” n.d. Accessed August 8, 2018. https://link.springer.com/article/10.1007/s11356-015-5670-5. |
| 11 | fruits | apple | 0.360 | China | Yue, Qian, Xiangrui Xu, Jonathan Hillier, Kun Cheng, and Genxing Pan. 2017. “Mitigating Greenhouse Gas Emissions in Agriculture: From Farm Production to Food Consumption.” Journal of Cleaner Production 149 (April): 1011–19. https://doi.org/10.1016/j.jclepro.2017.02.172. |
| 11 | fruits | Chinese orange | 0.175 | China | Xu, Xiaoming, and Ying Lan. 2016. “A Comparative Study on Carbon Footprints between Plant- and Animal-Based Foods in China.” Journal of Cleaner Production 112 (January): 2581–92. https://doi.org/10.1016/j.jclepro.2015.10.059. |
| 11 | fruits | orange | 0.140 | China | “Farm and Product Carbon Footprints of China’s Fruit Production—Life Cycle Inventory of Representative Orchards of Five Major Fruits \| SpringerLink.” n.d. Accessed August 8, 2018. https://link.springer.com/article/10.1007/s11356-015-5670-5. |
| 11 | fruits | citrus | 0.310 | China | Yue, Qian, Xiangrui Xu, Jonathan Hillier, Kun Cheng, and Genxing Pan. 2017. “Mitigating Greenhouse Gas Emissions in Agriculture: From Farm Production to Food Consumption.” Journal of Cleaner Production 149 (April): 1011–19. https://doi.org/10.1016/j.jclepro.2017.02.172. |
| 11 | fruits | tangerine | 0.240 | China | Yue, Qian, Xiangrui Xu, Jonathan Hillier, Kun Cheng, and Genxing Pan. 2017. “Mitigating Greenhouse Gas Emissions in Agriculture: From Farm Production to Food Consumption.” Journal of Cleaner Production 149 (April): 1011–19. https://doi.org/10.1016/j.jclepro.2017.02.172. |
| 11 | fruits | tangerine | 0.119 | China | Xu, Xiaoming, and Ying Lan. 2016. “A Comparative Study on Carbon Footprints between Plant- and Animal-Based Foods in China.” Journal of Cleaner Production 112 (January): 2581–92. https://doi.org/10.1016/j.jclepro.2015.10.059. |
| 11 | fruits | banana | 0.270 | China | “Farm and Product Carbon Footprints of China’s Fruit Production—Life Cycle Inventory of Representative Orchards of Five Major Fruits \| SpringerLink.” n.d. Accessed August 8, 2018. https://link.springer.com/article/10.1007/s11356-015-5670-5. |
| 11 | fruits | peach | 0.370 | China | “Farm and Product Carbon Footprints of China’s Fruit Production—Life Cycle Inventory of Representative Orchards of Five Major Fruits \| SpringerLink.” n.d. Accessed August 8, 2018. https://link.springer.com/article/10.1007/s11356-015-5670-5. |
| 11 | fruit | peach | 0.317 | China (three regions) | Guo, C., Wang, X., Li, Y., He, X., Zhang, W., Wang, J., Shi, X., Chen, X., Zhang, Y., 2018. Carbon Footprint Analyses and Potential Carbon Emission Reduction in China’s Major Peach Orchards. Sustainability 10, 2908. https://doi.org/10.3390/su10082908 |
| 11 | fruits | pear | 0.180 | China | “Farm and Product Carbon Footprints of China’s Fruit Production—Life Cycle Inventory of Representative Orchards of Five Major Fruits \| SpringerLink.” n.d. Accessed August 8, 2018. https://link.springer.com/article/10.1007/s11356-015-5670-5. |
| 11 | fruit | pear | 0.220 | China (5 provinces) | Liu, Y., Langer, V., Høgh-Jensen, H., Egelyng, H., 2010. Life Cycle Assessment of fossil energy use and greenhouse gas emissions in Chinese pear production. J. Clean. Prod. 18, 1423–1430. https://doi.org/10.1016/j.jclepro.2010.05.025 |
| 11 | fruits | grapes | 0.620 | Industrialized Asia: China, Japan, South Korea | Porter, Stephen D., David S. Reay, Peter Higgins, and Elizabeth Bomberg. 2016. “A Half-Century of Production-Phase Greenhouse Gas Emissions from Food Loss & Waste in the Global Food Supply Chain.” Science of The Total Environment 571 (November): 721–29. https://doi.org/10.1016/j.scitotenv.2016.07.041. |
| 11 | fruits | fruit average | 0.890 | China | Jianyi, Lin, Hu Yuanchao, Cui Shenghui, Kang Jiefeng, and Xu Lilai. 2015. “Carbon Footprints of Food Production in China (1979–2009).” Journal of Cleaner Production 90 (March): 97–103. https://doi.org/10.1016/j.jclepro.2014.11.072. |
| 11 | fruits | fruit average | 0.310 | China | Yue, Qian, Xiangrui Xu, Jonathan Hillier, Kun Cheng, and Genxing Pan. 2017. “Mitigating Greenhouse Gas Emissions in Agriculture: From Farm Production to Food Consumption.” Journal of Cleaner Production 149 (April): 1011–19. https://doi.org/10.1016/j.jclepro.2017.02.172. |
| 12 | nuts and seeds | peanut | 0.489 | China | Xu, Xiaoming, and Ying Lan. 2016. “A Comparative Study on Carbon Footprints between Plant- and Animal-Based Foods in China.” Journal of Cleaner Production 112 (January): 2581–92. https://doi.org/10.1016/j.jclepro.2015.10.059. |
| 12 | nuts and seeds | peanut | 0.900 | China | Yue, Qian, Xiangrui Xu, Jonathan Hillier, Kun Cheng, and Genxing Pan. 2017. “Mitigating Greenhouse Gas Emissions in Agriculture: From Farm Production to Food Consumption.” Journal of Cleaner Production 149 (April): 1011–19. https://doi.org/10.1016/j.jclepro.2017.02.172. |
| 12 | nuts and seeds | sunflower | 0.079 | China | Liu, Weiwei, Guo Zhang, Xiaoke Wang, Fei Lu, and Zhiyun Ouyang. 2018. “Carbon Footprint of Main Crop Production in China: Magnitude, Spatial-Temporal Pattern and Attribution.” Science of The Total Environment 645 (December): 1296–1308. https://doi.org/10.1016/j.scitotenv.2018.07.104. |
| 12 | nuts and seeds | walnuts | 1.147 | France | Vieux, F., N. Darmon, D. Touazi, and L. G. Soler. 2012. “Greenhouse Gas Emissions of Self-Selected Individual Diets in France: Changing the Diet Structure or Consuming Less?” Ecological Economics 75 (C): 91–101. |
| 13 | pork | pork | 2.150 | China | Xu, Xiaoming, and Ying Lan. 2016. “A Comparative Study on Carbon Footprints between Plant- and Animal-Based Foods in China.” Journal of Cleaner Production 112 (January): 2581–92. https://doi.org/10.1016/j.jclepro.2015.10.059. |
| 13 | pork | pork | 4.840 | China Sichuan province | Luo, Ting, Qian Yue, Ming Yan, Kun Cheng, and Genxing Pan. 2015. “Carbon Footprint of China’s Livestock System – a Case Study of Farm Survey in Sichuan Province, China.” Journal of Cleaner Production 102 (September): 136–43. https://doi.org/10.1016/j.jclepro.2015.04.077. |
| 13 | pork | pork | 1.140 | China | Wang, Li-zhi, Bai Xue, and Tianhai Yan. 2017. “Greenhouse Gas Emissions from Pig and Poultry Production Sectors in China from 1960 to 2010.” Journal of Integrative Agriculture 16 (1): 221–28. https://doi.org/10.1016/S2095-3119(16)61372-2. |
| 13 | pork | pork | 3.390 | Northern China (Hebei) | Zhou, Y., Dong, H., Xin, H., Zhu, Z., Huang, W., Wang, Y., 2018. Carbon Footprint Assessment of a Large-Scale Pig Production System in Northern China: A Case Study. Trans. ASABE 61, 1121–1131. https://doi.org/10.13031/trans.12805 |
| 13 | pork | pork | 2.890 | China | Jianyi, Lin, Hu Yuanchao, Cui Shenghui, Kang Jiefeng, and Xu Lilai. 2015. “Carbon Footprints of Food Production in China (1979–2009).” Journal of Cleaner Production 90 (March): 97–103. https://doi.org/10.1016/j.jclepro.2014.11.072. |
| 13 | pork | pork | 6.183 | China | Yue, Qian, Xiangrui Xu, Jonathan Hillier, Kun Cheng, and Genxing Pan. 2017. “Mitigating Greenhouse Gas Emissions in Agriculture: From Farm Production to Food Consumption.” Journal of Cleaner Production 149 (April): 1011–19. https://doi.org/10.1016/j.jclepro.2017.02.172. |
| 14 | beef | beef | 21.710 | China | Jianyi, Lin, Hu Yuanchao, Cui Shenghui, Kang Jiefeng, and Xu Lilai. 2015. “Carbon Footprints of Food Production in China (1979–2009).” Journal of Cleaner Production 90 (March): 97–103. https://doi.org/10.1016/j.jclepro.2014.11.072. |
| 14 | beef | beef | 8.310 | China | Xu, Xiaoming, and Ying Lan. 2016. “A Comparative Study on Carbon Footprints between Plant- and Animal-Based Foods in China.” Journal of Cleaner Production 112 (January): 2581–92. https://doi.org/10.1016/j.jclepro.2015.10.059. |
| 14 | beef | beef | 7.300 | China | Yue, Qian, Xiangrui Xu, Jonathan Hillier, Kun Cheng, and Genxing Pan. 2017. “Mitigating Greenhouse Gas Emissions in Agriculture: From Farm Production to Food Consumption.” Journal of Cleaner Production 149 (April): 1011–19. https://doi.org/10.1016/j.jclepro.2017.02.172. |
| 15 | mutton and goat | mutton and goat | 20.820 | China | Jianyi, Lin, Hu Yuanchao, Cui Shenghui, Kang Jiefeng, and Xu Lilai. 2015. “Carbon Footprints of Food Production in China (1979–2009).” Journal of Cleaner Production 90 (March): 97–103. https://doi.org/10.1016/j.jclepro.2014.11.072. |
| 15 | mutton and goat | mutton | 8.340 | China | Xu, Xiaoming, and Ying Lan. 2016. “A Comparative Study on Carbon Footprints between Plant- and Animal-Based Foods in China.” Journal of Cleaner Production 112 (January): 2581–92. https://doi.org/10.1016/j.jclepro.2015.10.059. |
| 15 | mutton and goat | mutton | 8.920 | China | Yue, Qian, Xiangrui Xu, Jonathan Hillier, Kun Cheng, and Genxing Pan. 2017. “Mitigating Greenhouse Gas Emissions in Agriculture: From Farm Production to Food Consumption.” Journal of Cleaner Production 149 (April): 1011–19. https://doi.org/10.1016/j.jclepro.2017.02.172. |
| 17 | poultry | chicken | 2.238 | China | Xu, Xiaoming, and Ying Lan. 2016. “A Comparative Study on Carbon Footprints between Plant- and Animal-Based Foods in China.” Journal of Cleaner Production 112 (January): 2581–92. https://doi.org/10.1016/j.jclepro.2015.10.059. |
| 17 | poultry | chicken | 13.940 | China Sichuan province | Luo, Ting, Qian Yue, Ming Yan, Kun Cheng, and Genxing Pan. 2015. “Carbon Footprint of China’s Livestock System – a Case Study of Farm Survey in Sichuan Province, China.” Journal of Cleaner Production 102 (September): 136–43. https://doi.org/10.1016/j.jclepro.2015.04.077. |
| 17 | poultry | chicken | 5.260 | China | Yue, Qian, Xiangrui Xu, Jonathan Hillier, Kun Cheng, and Genxing Pan. 2017. “Mitigating Greenhouse Gas Emissions in Agriculture: From Farm Production to Food Consumption.” Journal of Cleaner Production 149 (April): 1011–19. https://doi.org/10.1016/j.jclepro.2017.02.172. |
| 17 | poultry | poultry | 1.130 | China | Jianyi, Lin, Hu Yuanchao, Cui Shenghui, Kang Jiefeng, and Xu Lilai. 2015. “Carbon Footprints of Food Production in China (1979–2009).” Journal of Cleaner Production 90 (March): 97–103. https://doi.org/10.1016/j.jclepro.2014.11.072. |
| 17 | poultry | poultry | 0.370 | China | Wang, Li-zhi, Bai Xue, and Tianhai Yan. 2017. “Greenhouse Gas Emissions from Pig and Poultry Production Sectors in China from 1960 to 2010.” Journal of Integrative Agriculture 16 (1): 221–28. https://doi.org/10.1016/S2095-3119(16)61372-2. |
| 19 | milk | milk | 0.951 | China | Xu, Xiaoming, and Ying Lan. 2016. “A Comparative Study on Carbon Footprints between Plant- and Animal-Based Foods in China.” Journal of Cleaner Production 112 (January): 2581–92. https://doi.org/10.1016/j.jclepro.2015.10.059. |
| 19 | milk | milk | 1.470 | China | Yue, Qian, Xiangrui Xu, Jonathan Hillier, Kun Cheng, and Genxing Pan. 2017. “Mitigating Greenhouse Gas Emissions in Agriculture: From Farm Production to Food Consumption.” Journal of Cleaner Production 149 (April): 1011–19. https://doi.org/10.1016/j.jclepro.2017.02.172. |
| 19 | milk | milk | 1.070 | China Sichuan province | Luo, Ting, Qian Yue, Ming Yan, Kun Cheng, and Genxing Pan. 2015. “Carbon Footprint of China’s Livestock System – a Case Study of Farm Survey in Sichuan Province, China.” Journal of Cleaner Production 102 (September): 136–43. https://doi.org/10.1016/j.jclepro.2015.04.077. |
| 19 | milk | milk | 1.650 | China | Jianyi, Lin, Hu Yuanchao, Cui Shenghui, Kang Jiefeng, and Xu Lilai. 2015. “Carbon Footprints of Food Production in China (1979–2009).” Journal of Cleaner Production 90 (March): 97–103. https://doi.org/10.1016/j.jclepro.2014.11.072. |
| 21 | eggs | eggs | 1.238 | China | Xu, Xiaoming, and Ying Lan. 2016. “A Comparative Study on Carbon Footprints between Plant- and Animal-Based Foods in China.” Journal of Cleaner Production 112 (January): 2581–92. https://doi.org/10.1016/j.jclepro.2015.10.059. |
| 21 | eggs | eggs | 1.140 | China | Jianyi, Lin, Hu Yuanchao, Cui Shenghui, Kang Jiefeng, and Xu Lilai. 2015. “Carbon Footprints of Food Production in China (1979–2009).” Journal of Cleaner Production 90 (March): 97–103. https://doi.org/10.1016/j.jclepro.2014.11.072. |
| 21 | eggs | eggs | 0.330 | China | Wang, Li-zhi, Bai Xue, and Tianhai Yan. 2017. “Greenhouse Gas Emissions from Pig and Poultry Production Sectors in China from 1960 to 2010.” Journal of Integrative Agriculture 16 (1): 221–28. https://doi.org/10.1016/S2095-3119(16)61372-2. |
| 21 | eggs | poultry eggs | 4.090 | China | Yue, Qian, Xiangrui Xu, Jonathan Hillier, Kun Cheng, and Genxing Pan. 2017. “Mitigating Greenhouse Gas Emissions in Agriculture: From Farm Production to Food Consumption.” Journal of Cleaner Production 149 (April): 1011–19. https://doi.org/10.1016/j.jclepro.2017.02.172. |
| 21 | eggs | eggs | 3.580 | China Sichuan province | Luo, Ting, Qian Yue, Ming Yan, Kun Cheng, and Genxing Pan. 2015. “Carbon Footprint of China’s Livestock System – a Case Study of Farm Survey in Sichuan Province, China.” Journal of Cleaner Production 102 (September): 136–43. https://doi.org/10.1016/j.jclepro.2015.04.077. |
| 22 | fish | fish and seafood | 2.770 | Industrialized Asia: China, Japan, South Korea | Porter, Stephen D., David S. Reay, Peter Higgins, and Elizabeth Bomberg. 2016. “A Half-Century of Production-Phase Greenhouse Gas Emissions from Food Loss & Waste in the Global Food Supply Chain.” Science of The Total Environment 571 (November): 721–29. https://doi.org/10.1016/j.scitotenv.2016.07.041. |
| 23 | juice | pasteurised orange juice | 1.147 | France | Vieux, F., N. Darmon, D. Touazi, and L. G. Soler. 2012. “Greenhouse Gas Emissions of Self-Selected Individual Diets in France: Changing the Diet Structure or Consuming Less?” *Ecological Economics* 75 (C): 91–101. |
| 24 | other drinks | black coffee | 0.363 | France | Vieux, F., N. Darmon, D. Touazi, and L. G. Soler. 2012. “Greenhouse Gas Emissions of Self-Selected Individual Diets in France: Changing the Diet Structure or Consuming Less?” Ecological Economics 75 (C): 91–101. |
| 24 | other drinks | tea | 0.059 | France | Vieux, F., N. Darmon, D. Touazi, and L. G. Soler. 2012. “Greenhouse Gas Emissions of Self-Selected Individual Diets in France: Changing the Diet Structure or Consuming Less?” Ecological Economics 75 (C): 91–101. |
| 24 | other drinks | spring water | 0.175 | France | Vieux, F., N. Darmon, D. Touazi, and L. G. Soler. 2012. “Greenhouse Gas Emissions of Self-Selected Individual Diets in France: Changing the Diet Structure or Consuming Less?” Ecological Economics 75 (C): 91–101. |
| 25 | high sugar drinks | soda | 0.363 | France | Vieux, F., N. Darmon, D. Touazi, and L. G. Soler. 2012. “Greenhouse Gas Emissions of Self-Selected Individual Diets in France: Changing the Diet Structure or Consuming Less?” Ecological Economics 75 (C): 91–101. |
| 26 | processed foods | crackers | 2.488 | France | Vieux, F., N. Darmon, D. Touazi, and L. G. Soler. 2012. “Greenhouse Gas Emissions of Self-Selected Individual Diets in France: Changing the Diet Structure or Consuming Less?” Ecological Economics 75 (C): 91–101. |
| 26 | processed foods | salted potato chips | 2.586 | France | Vieux, F., N. Darmon, D. Touazi, and L. G. Soler. 2012. “Greenhouse Gas Emissions of Self-Selected Individual Diets in France: Changing the Diet Structure or Consuming Less?” Ecological Economics 75 (C): 91–101. |
| 26 | processed foods | cheeseburger | 7.451 | France | Vieux, F., N. Darmon, D. Touazi, and L. G. Soler. 2012. “Greenhouse Gas Emissions of Self-Selected Individual Diets in France: Changing the Diet Structure or Consuming Less?” Ecological Economics 75 (C): 91–101. |
| 26 | processed foods | pizza | 4.484 | France | Vieux, F., N. Darmon, D. Touazi, and L. G. Soler. 2012. “Greenhouse Gas Emissions of Self-Selected Individual Diets in France: Changing the Diet Structure or Consuming Less?” Ecological Economics 75 (C): 91–101. |
| 27 | sweets | cane sugar | 0.335 | China | Xu, Xiaoming, and Ying Lan. 2016. “A Comparative Study on Carbon Footprints between Plant- and Animal-Based Foods in China.” Journal of Cleaner Production 112 (January): 2581–92. https://doi.org/10.1016/j.jclepro.2015.10.059. |
| 27 | sweets | beet sugar | 0.495 | China | Xu, Xiaoming, and Ying Lan. 2016. “A Comparative Study on Carbon Footprints between Plant- and Animal-Based Foods in China.” Journal of Cleaner Production 112 (January): 2581–92. https://doi.org/10.1016/j.jclepro.2015.10.059. |
| 27 | sweets | sugar average | 0.060 | China | Jianyi, Lin, Hu Yuanchao, Cui Shenghui, Kang Jiefeng, and Xu Lilai. 2015. “Carbon Footprints of Food Production in China (1979–2009).” Journal of Cleaner Production 90 (March): 97–103. https://doi.org/10.1016/j.jclepro.2014.11.072. |
| 27 | sweets | pain au chocolat | 2.047 | France | Vieux, F., N. Darmon, D. Touazi, and L. G. Soler. 2012. “Greenhouse Gas Emissions of Self-Selected Individual Diets in France: Changing the Diet Structure or Consuming Less?” Ecological Economics 75 (C): 91–101. |
| 27 | sweets | brioche | 2.625 | France | Vieux, F., N. Darmon, D. Touazi, and L. G. Soler. 2012. “Greenhouse Gas Emissions of Self-Selected Individual Diets in France: Changing the Diet Structure or Consuming Less?” Ecological Economics 75 (C): 91–101. |
| 27 | sweets | pie or fruit tart | 4.360 | France | Vieux, F., N. Darmon, D. Touazi, and L. G. Soler. 2012. “Greenhouse Gas Emissions of Self-Selected Individual Diets in France: Changing the Diet Structure or Consuming Less?” Ecological Economics 75 (C): 91–101. |
| 27 | sweets | honey | 1.008 | France | Vieux, F., N. Darmon, D. Touazi, and L. G. Soler. 2012. “Greenhouse Gas Emissions of Self-Selected Individual Diets in France: Changing the Diet Structure or Consuming Less?” Ecological Economics 75 (C): 91–101. |
| 28 | fats and oils | soybean oil | 0.933 | China | Xu, Xiaoming, and Ying Lan. 2016. “A Comparative Study on Carbon Footprints between Plant- and Animal-Based Foods in China.” Journal of Cleaner Production 112 (January): 2581–92. https://doi.org/10.1016/j.jclepro.2015.10.059. |
| 28 | fats and oils | peanut oil | 0.798 | China | Xu, Xiaoming, and Ying Lan. 2016. “A Comparative Study on Carbon Footprints between Plant- and Animal-Based Foods in China.” Journal of Cleaner Production 112 (January): 2581–92. https://doi.org/10.1016/j.jclepro.2015.10.059. |
| 28 | fats and oils | rapeseed oil | 1.646 | China | Xu, Xiaoming, and Ying Lan. 2016. “A Comparative Study on Carbon Footprints between Plant- and Animal-Based Foods in China.” Journal of Cleaner Production 112 (January): 2581–92. https://doi.org/10.1016/j.jclepro.2015.10.059. |

***Table 2.*** *Sources of emissions data*

| Emissions from production and at each post-production stage (g of CO_2_ eq. per 100 grams of food consumed) | | | | | | | | | | | | | |
| --- | --- | --- | --- | --- | --- | --- | --- | --- | --- | --- | --- | --- | --- |
|  |  |  |  | **% waste and losses at different stages** | | | | | **post-production emissions** | | | |  |
| **group  #** | **food group** | **individual  foods** | **production  GHGE (kg of CO_2_ eq. per kg)** | **agricultural production** | **postharvest handling  and storage** | **processing and  packaging** | **distribution** | **consumption** | **processing  and packaging** | **storage** | **transportation  (from farm to store)** | **cooking** | **total emissions  after cooking (kg of CO_2_ per kg of food consumed)** |
| 1 | wheat | **wheat grain** | 0.421 | 2% | 10.0% | 10.5% | 2.0% | 20.0% | 0.1 | 0.04 | 0.03 | 0.04 | 0.858 |
| 1 | wheat | **bread** | 0.784 | 2% | 10.0% | 10.5% | 2.0% | 20.0% | 0.1 | 0.0035 | 0.03 | 0.00 | 1.333 |
| 1 | wheat | **flour** | 0.784 | 2% | 10.0% | 10.5% | 2.0% | 20.0% | 0.1 | 0.04 | 0.03 | 0.04 | 1.400 |
| 2 | rice | **rice** | 1.054 | 2% | 10.0% | 10.5% | 2.0% | 20.0% | 0.16 | 0.04 | 0.03 | 0.04 | 1.875 |
| 3 | maize | **maize** | 0.332 | 2% | 10.0% | 10.5% | 2.0% | 20.0% | 0.1 | 0.04 | 0.03 | 0.04 | 0.725 |
| 4 | other cereals | **barley** | 0.630 | 2% | 10.0% | 10.5% | 2.0% | 20.0% | 0.1 | 0.04 | 0.03 | 0.04 | 1.170 |
| 4 | other cereals | **millet** | 0.930 | 2% | 10.0% | 10.5% | 2.0% | 20.0% | 0.1 | 0.04 | 0.03 | 0.04 | 1.618 |
| 4 | other cereals | **oats** | 0.930 | 2% | 10.0% | 10.5% | 2.0% | 20.0% | 0.1 | 0.04 | 0.03 | 0.04 | 1.618 |
| 4 | other cereals | **sorghum** | 0.367 | 2% | 10.0% | 10.5% | 2.0% | 20.0% | 0.1 | 0.04 | 0.03 | 0.04 | 0.777 |
| 4 | other cereals | **other cereals** | 0.930 | 2% | 10.0% | 10.5% | 2.0% | 20.0% | 0.1 | 0.04 | 0.03 | 0.04 | 1.618 |
| 5 | tubers | **starchy roots** | 0.190 | 20% | 7.0% | 15.0% | 9.0% | 10.0% | 0 | 0 | 0.03 | 0.25 | 0.487 |
| 5 | tubers | **potato** | 0.107 | 20% | 7.0% | 15.0% | 9.0% | 10.0% | 0 | 0 | 0.03 | 0.25 | 0.349 |
| 6 | soybeans | **soybeans** | 0.326 | 6% | 3% | 5% | 1% | 4% | 0 | 0.04 | 0.03 | 0.24 | 0.588 |
| 7 | beans and pulses | **pulses** | 0.349 | 6% | 3% | 5% | 1% | 4% | 0 | 0.04 | 0.03 | 0.24 | 0.616 |
| 7 | beans and pulses | **kidney beans** | 0.190 | 6% | 3% | 5% | 1% | 4% | 0 | 0.04 | 0.03 | 0.24 | 0.426 |
| 8 | vegetables | **tomato** | 0.089 | 10% | 8.0% | 2.0% | 8.0% | 15.0% | 0 | 0.02 | 0.03 | 0.05 | 0.218 |
| 8 | vegetables | **radish** | 0.029 | 10% | 8.0% | 2.0% | 8.0% | 15.0% | 0 | 0.02 | 0.03 | 0.05 | 0.129 |
| 8 | vegetables | **cucumber** | 0.080 | 10% | 8.0% | 2.0% | 8.0% | 15.0% | 0 | 0.02 | 0.03 | 0.05 | 0.204 |
| 8 | vegetables | **eggplant** | 0.100 | 10% | 8.0% | 2.0% | 8.0% | 15.0% | 0 | 0.02 | 0.03 | 0.05 | 0.234 |
| 8 | vegetables | **green pepper** | 0.225 | 10% | 8.0% | 2.0% | 8.0% | 15.0% | 0 | 0.02 | 0.03 | 0.05 | 0.418 |
| 8 | vegetables | **green beans** | 0.060 | 10% | 8.0% | 2.0% | 8.0% | 15.0% | 0 | 0.02 | 0.03 | 0.05 | 0.175 |
| 9 | leafy greens | **cabbage** | 0.123 | 10% | 8.0% | 2.0% | 8.0% | 15.0% | 0 | 0.02 | 0.03 | 0.05 | 0.267 |
| 9 | leafy greens | **Chinese cabbage** | 0.058 | 10% | 8.0% | 2.0% | 8.0% | 15.0% | 0 | 0.02 | 0.03 | 0.05 | 0.172 |
| 9 | leafy greens | **cauliflower** | 0.132 | 10% | 8.0% | 2.0% | 8.0% | 15.0% | 0 | 0.02 | 0.03 | 0.05 | 0.282 |
| 10 | mushrooms | **mushrooms** | 0.730 | 10% | 8.0% | 2.0% | 8.0% | 15.0% | 0 | 0.02 | 0.03 | 0.05 | 1.162 |
| 11 | fruit | **apple** | 0.222 | 10% | 8.0% | 2.0% | 8.0% | 15.0% | 0 | 0.02 | 0.03 | 0.00 | 0.386 |
| 11 | fruit | **Chinese orange** | 0.156 | 10% | 8.0% | 2.0% | 8.0% | 15.0% | 0 | 0.02 | 0.03 | 0.00 | 0.288 |
| 11 | fruit | **citrus** | 0.310 | 10% | 8.0% | 2.0% | 8.0% | 15.0% | 0 | 0.02 | 0.03 | 0.00 | 0.515 |
| 11 | fruit | **tangerine** | 0.169 | 10% | 8.0% | 2.0% | 8.0% | 15.0% | 0 | 0.02 | 0.03 | 0.00 | 0.307 |
| 11 | fruit | **banana** | 0.270 | 10% | 8.0% | 2.0% | 8.0% | 15.0% | 0 | 0.02 | 0.03 | 0.00 | 0.456 |
| 11 | fruit | **peach** | 0.342 | 10% | 8.0% | 2.0% | 8.0% | 15.0% | 0 | 0.02 | 0.03 | 0.00 | 0.562 |
| 11 | fruit | **pear** | 0.199 | 10% | 8.0% | 2.0% | 8.0% | 15.0% | 0 | 0.02 | 0.03 | 0.00 | 0.351 |
| 11 | fruit | **grapes** | 0.620 | 10% | 8.0% | 2.0% | 8.0% | 15.0% | 0 | 0.02 | 0.03 | 0.00 | 0.971 |
| 12 | nuts and seeds | **peanut** | 0.663 | 6% | 3% | 5% | 1% | 4% | 0 | 0.04 | 0.03 | 0.00 | 0.867 |
| 12 | nuts and seeds | **sunflower** | 0.079 | 6% | 3% | 5% | 1% | 4% | 0 | 0.04 | 0.03 | 0.00 | 0.168 |
| 12 | nuts and seeds | **walnuts** | 1.147 | 0% | 0% | 0% | 0% | 4% | 0 | 0.04 | 0 | 0.00 | 1.235 |
| 13 | pork | **pork** | 4.180 | 2.90% | 0.6% | 5.0% | 6.0% | 8.0% | 0 | 0.002 | 0.03 | 0.24 | 5.333 |
| 14 | beef | **beef** | 14.467 | 2.90% | 0.6% | 5.0% | 6.0% | 8.0% | 0 | 0.0015 | 0.03 | 0.24 | 18.054 |
| 15 | mutton | **mutton and goat** | 12.438 | 2.90% | 0.6% | 5.0% | 6.0% | 8.0% | 0 | 0.0021 | 0.03 | 0.26 | 15.556 |
| 16 | other red meat | **average of beef, lamb, pork** | 10.361 | 2.90% | 0.6% | 5.0% | 6.0% | 8.0% | 0 | 0.0019 | 0.03 | 0.25 | 12.981 |
| 17 | poultry | **chicken** | 6.670 | 2.90% | 0.6% | 5.0% | 6.0% | 8.0% | 0 | 0.002 | 0.03 | 0.27 | 8.429 |
| 18 | other white meat | **chicken values assumed** | 6.670 | 2.90% | 0.6% | 5.0% | 6.0% | 8.0% | 0 | 0.002 | 0.03 | 0.27 | 8.429 |
| 19 | milk and milk powder | **milk** | 1.255 | 3.50% | 1.0% | 1.2% | 0.5% | 5.0% | 0.12 | 0 | 0.03 | 0.00 | 1.557 |
| 19 | milk and milk powder | **powder milk** | 3.802 | 3.50% | 1.0% | 1.2% | 0.5% | 5.0% | 0.12 | 0 | 0.03 | 0.00 | 4.398 |
| 20 | other dairy | **yoghurt** | 1.255 | 3.50% | 1.0% | 1.2% | 0.5% | 5.0% | 0.12 | 0 | 0.03 | 0.00 | 1.557 |
| 20 | other dairy | **cheese** | 8.365 | 3.50% | 1.0% | 1.2% | 0.5% | 5.0% | 1 | 0.01 | 0.03 | 0.00 | 10.420 |
| 20 | other dairy | **butter** | 31.368 | 3.50% | 1.0% | 1.2% | 0.5% | 5.0% | 0.66 | 0.01 | 0.03 | 0.00 | 35.714 |
| 20 | other dairy | **condensed milk** | 3.302 | 3.50% | 1.0% | 1.2% | 0.5% | 5.0% | 1 | 0.01 | 0.03 | 0.00 | 4.775 |
| 20 | other dairy | **ice-cream** | 1.255 | 3.50% | 1.0% | 1.2% | 0.5% | 5.0% | 1 | 0.01 | 0.03 | 0.00 | 2.492 |
| 21 | eggs | **eggs** | 2.463 | 3.50% | 1.0% | 1.2% | 0.5% | 5.0% | 0 | 0 | 0.03 | 0.10 | 2.832 |
| 22 | fish and seafood | **fish and seafood** | 2.770 | 15% | 2% | 6% | 11% | 8% | 0.1 | 0.000 | 0.03 | 0.09 | 4.200 |
| 23 | juice (fruit and veg) | **juice** | 1.147 | 0% | 0% | 0% | 0% | 0% | 0 | 0.002 | 0 | 0.00 | 1.150 |
| 24 | other drinks (coffee, tea, water) | **black coffee** | 0.363 | 0% | 0% | 0% | 0% | 0% | 0 | 0.037 | 0 | 0.02 | 0.409 |
| 24 | other drinks (coffee, tea, water) | **tea** | 0.059 | 0% | 0% | 0% | 0% | 0% | 0 | 0.037 | 0 | 0.02 | 0.105 |
| 24 | other drinks (coffee, tea, water) | **spring water** | 0.175 | 0% | 0% | 0% | 0% | 0% | 0 | 0.030 | 0 | 0.00 | 0.206 |
| 25 | high sugar drinks | **soda** | 0.363 | 0% | 0% | 0% | 0% | 0% | 0 | 0.030 | 0 | 0.00 | 0.394 |
| 26 | processed foods | **crackers** | 2.488 | 0% | 0% | 0% | 0% | 0% | 0 | 0.030 | 0 | 0.00 | 2.519 |
| 26 | processed foods | **salted potato chips** | 2.586 | 0% | 0% | 0% | 0% | 0% | 0 | 0.030 | 0 | 0.00 | 2.617 |
| 26 | processed foods | **cheeseburger** | 7.451 | 0% | 0% | 0% | 0% | 0% | 0 | 0.030 | 0 | 0.00 | 7.482 |
| 26 | processed foods | **pizza** | 4.484 | 0% | 0% | 0% | 0% | 0% | 0 | 0.030 | 0 | 0.00 | 4.514 |
| 27 | sweets | **cane sugar** | 0.335 | 10% | 8.0% | 2.0% | 8.0% | 15.0% | 0.1 | 0.037 | 0.03 | 0.00 | 0.685 |
| 27 | sweets | **beet sugar** | 0.495 | 10% | 8.0% | 2.0% | 8.0% | 15.0% | 0.1 | 0.037 | 0.03 | 0.00 | 0.921 |
| 27 | sweets | **honey** | 1.008 | 0% | 0% | 0% | 0% | 0% | 0 | 0.037 | 0 | 0.00 | 1.045 |
| 27 | sweets | **pain au chocolat** | 2.047 | 0% | 0% | 0% | 0% | 0% | 0 | 0.030 | 0 | 0.00 | 2.078 |
| 27 | sweets | **brioche** | 2.625 | 0% | 0% | 0% | 0% | 0% | 0 | 0.030 | 0 | 0.00 | 2.655 |
| 27 | sweets | **pie or fruit tart** | 4.360 | 0% | 0% | 0% | 0% | 0% | 0 | 0.030 | 0.03 | 0.00 | 4.421 |
| 28 | fats and oils | **soybean oil** | 0.933 | 6% | 3% | 5% | 1% | 4% | 0.16 | 0.02 | 0.03 | 0.00 | 1.334 |
| 28 | fats and oils | **peanut oil** | 0.798 | 6% | 3% | 5% | 1% | 4% | 0.16 | 0.02 | 0.03 | 0.00 | 1.173 |
| 28 | fats and oils | **rapeseed oil** | 1.646 | 6% | 3% | 5% | 1% | 4% | 0.16 | 0.02 | 0.03 | 0.00 | 2.187 |

***Table 3.*** *Emissions from production and at each post-production stage*

**Appendix C – WHO constraints in equation form**

| **WHO constraints** | | **Our constraints** |
| --- | --- | --- |
| **Simulation model 1** | | |
| Dietary factor | Goal (% of total energy, unless otherwise stated) |  |
| Total fat | 15-30% | $0.15\sum_{i\in F} e_{i}Q_{i}^{'}\leq9\sum_{i\in F} f_{i}Q_{i}^{'}\leq0.3\sum_{i\in F} e_{i}Q_{i}^{'}$ |
| Saturated fatty acids | <10% | Not modelled due to lack of data |
| Polyunsaturated fatty acids (PUFAs) | 6-10% | Not modelled due to lack of data |
| n-6 polyunsaturated fatty acids (PUFAs) | 5-8% | Not modelled due to lack of data |
| n-3 polyunsaturated fatty acids (PUFAs) | 1-2% | Not modelled due to lack of data |
| Trans fatty acids | <1% | Not modelled due to lack of data |
| Monounsaturated fatty acids (MUFAs) | By difference | Not modelled due to lack of data |
| Total carbohydrate | 55-75% | Always true when protein and fat constraints are satisfied |
| Free sugars | <10% | $4\sum_{i\in F} s_{i}Q_{i}^{'}\leq0.1\sum_{i\in F} e_{i}Q_{i}^{'}$ |
| Protein | 10-15% | $0.1\sum_{i\in F} e_{i}Q_{i}^{'}\leq4\sum_{i\in F} p_{i}Q_{i}^{'}\leq0.15\sum_{i\in F} e_{i}Q_{i}^{'}$ |
| Cholesterol | <300 mg per day | Not modelled due to lack of data |
| Sodium chloride (sodium) | $\leq$2 g per day | $\sum_{i\in F} n_{i}Q_{i}^{'}\leq2$ |
| Fruits and vegetables | $\geq$400 g per day | $400\leq\sum_{i\in V} Q_{i}^{'}$ |
| Only positive amounts consumed* | | $Q_{i}^{'}\geq0$ |
| Caloric intake in simulated diet is the same as current caloric  Intake level* | | $\sum_{i\in F} e_{i}Q_{i}=\sum_{i\in F} e_{i}Q_{i}^{'}$ |
| Budget constraint*  *constraints are not in the CDG recommendations | | $\sum_{i\in F} {{price}_{i}Q}_{i}^{'}\leq daily income$ |
| **Simulation model 2** – food, budget and GHGE constraints: includes all constraints from simulation 1 in addition to: | | |
| Cap on daily diet-related GHGEs | | $\sum_{i\in F} {ghge}_{i}Q_{i}^{'}\leq p\%\cdot\sum_{i\in F} {ghge}_{i}Q_{i}$ |

Table 4. WHO constraints

**Appendix D – CDG constraints in equation form**

| **CGD constraints** | **Our constraints** |
| --- | --- |
| **Simulation model 1** | |
| Salt <6g | $\sum_{i\in F} {n_{i}Q}_{i}^{'}\leq6$ |
| Cooking oil 25-30g | $25\leq\sum_{i\in Cooking oil} Q_{i}^{'}\leq30$ |
| Milk and dairy products 300g | $\sum_{i\in Dairy} Q_{i}^{'}=300$ |
| Soybeans and nuts 25-35g | $25\leq\sum_{i\in Soy\&Nuts} Q_{i}^{'}\leq35$ |
| Lean meats 40-75g | $40\leq\sum_{i\in Meat} Q_{i}^{'}\leq75$ |
| Fish 40-75g | $40\leq\sum_{i\in Fish} Q_{i}^{'}\leq75$ |
| Eggs 40-50g | $40\leq\sum_{i\in Eggs} Q_{i}^{'}\leq50$ |
| Vegetables 300-500g | $300\leq\sum_{i\in Veg} Q_{i}^{'}\leq500$ |
| Fruits 200-350g | $200\leq\sum_{i\in Fruit} Q_{i}^{'}\leq35$ |
| Cereals, tubers and legumes 250-400g | $250\leq\sum_{i\in Cereals, tubers, beans} Q_{i}^{'}\leq400$ |
| Whole grains and legumes 50-150g | Not modelled |
| Tubers 50-100g | Not modelled |
| Total energy 1600-2400Kcal | $1600\leq\sum_{i\in F} e_{i}Q_{i}^{'}\leq2400$ |
| Added sugar <25g | $\sum_{i\in processed foods} s_{i}Q_{i}^{'}\leq25$ |
| Only positive amounts consumed* | $Q_{i}^{'}\geq0$ |
| Budget constraint*  *Constraints are not in the CDG recommendations | $\sum_{i\in F} {P_{i}Q}_{i}^{'}\leq daily income$ |
| **Simulation model 2** – food, budget and GHGE constraints: includes all constraints from simulation 1 in addition to: | |
| Cap on daily diet-related GHGEs | $\sum_{i\in F} {ghge}_{i}Q_{i}^{'}\leq p\%\cdot\sum_{i\in F} {ghge}_{i}Q_{i}$ |

**Table 5.** CDG constraints

**Appendix E - A short introduction to diet-optimisation models**

Diet optimisation models assist in identifying sustainable diets (Gazan et al. 2018). Quadratic programming (QP) optimisation models consist of minimising the squared deviations from current consumption levels, under a number of constraints and assume consumers’ preferences and tastes are embodied in the initial food choices (Shankar et al. 2008). Extreme changes from the observed choices are improbable and, consequently, the deviations from the initial diet are minimised when estimating a new diet that conforms with dietary constraints (Green et al. 2015; Aleksandrowicz et al. 2019).

Environmental constraints are now becoming increasingly popular in QP models (Reynolds et al. 2019; Aleksandrowicz et al. 2019; Horgan et al. 2016). When exploring the environment-diets link, many authors (Springmann et al. 2018; Ritchie et al. 2018; Horgan et al. 2016) focus solely on one environmental aspect of food consumption and compute the GHGE associated with a variety of eating patterns. Oftentimes, the method of choice for determining carbon emissions involves life cycle assessment (LCA). LCA is used to estimate the GHGE linked with each food category, usually from “farm to fork” (Green et al. 2015; Aleksandrowicz et al. 2019; Green et al. 2018).

Making sure the proposed diets are agreeable to the public is essential since studies show the scale of dietary change that can be attained when aiming for a decrease in GHGE is highly correlated with the acceptability of the diet (Masset et al. 2014; Wilson et al. 2013). Horgan et al. (2016) and Reynolds et al. (2019) provided solutions to keeping dietary change to a minimum, by generating multiple diets limiting the deviation from the current dietary habits of each income quintile or individual. Green et al. (2015) simulated deviations in GHGE linked with UK consumers complying with the WHO dietary recommendations and considered potential substitutions between different food groups by incorporating in the analysis food price elasticities and expenditure shares. Their objective function constituted an approximation of consumers’ loss in welfare from adopting a new diet; minimising such an objective function ensured the resulting diets were realistic and appealing to consumers. Fu et al. (2024) employed multi-objective optimisation to model sustainable diets that are healthy and culturally acceptable but did not specifically focus on assessing the affordability of the proposed diets.

**References**

Aleksandrowicz, L., R. Green, E.J.M. Joy, F. Harris, J. Hillier, S.H. Vetter, P. Smith, B. Kulkarni, A.D. Dangour, and A. Haines. 2019. “Environmental Impacts of Dietary Shifts in India: A Modelling Study Using Nationally-Representative Data.” *Environment International* 126 (May):207–15. <https://doi.org/10.1016/j.envint.2019.02.004>.

Fu, Haiyue, Yating Li, Penghui Jiang, Shuai Zhou, and Chuan Liao. 2024. “Transition towards Sustainable Diets: Multi-Objective Optimization of Dietary Pattern in China.” *Sustainable Production and Consumption* 48 (July):14–28. <https://doi.org/10.1016/j.spc.2024.04.029>.

Gazan, Rozenn, Chloé M C Brouzes, Florent Vieux, Matthieu Maillot, Anne Lluch, and Nicole Darmon. 2018. “Mathematical Optimization to Explore Tomorrow’s Sustainable Diets: A Narrative Review.” *Advances in Nutrition* 9 (5): 602–16. <https://doi.org/10.1093/advances/nmy049>.

Green, Rosemary, James Milner, Alan D. Dangour, Andy Haines, Zaid Chalabi, Anil Markandya, Joseph Spadaro, and Paul Wilkinson. 2015. “The Potential to Reduce Greenhouse Gas Emissions in the UK through Healthy and Realistic Dietary Change.” *Climatic Change* 129 (1–2): 253–65. <https://doi.org/10.1007/s10584-015-1329-y>.

Horgan, Graham W., Amandine Perrin, Stephen Whybrow, and Jennie I. Macdiarmid. 2016. “Achieving Dietary Recommendations and Reducing Greenhouse Gas Emissions: Modelling Diets to Minimise the Change from Current Intakes.” *International Journal of Behavioral Nutrition & Physical Activity* 13 (April):1.

Masset, G., F. Vieux, E. O. Verger, L.-G. Soler, D. Touazi, and N. Darmon. 2014. “Reducing Energy Intake and Energy Density for a Sustainable Diet: A Study Based on Self-Selected Diets in French Adults.” *American Journal of Clinical Nutrition* 99 (6): 1460–69. <https://doi.org/10.3945/ajcn.113.077958>.

Reynolds, Christian J, Graham W Horgan, Stephen Whybrow, and Jennie I Macdiarmid. 2019. “Healthy and Sustainable Diets That Meet Greenhouse Gas Emission Reduction Targets and Are Affordable for Different Income Groups in the UK.” *Public Health Nutrition* 22 (8): 1503–17. <https://doi.org/10.1017/S1368980018003774>.

Ritchie, Hannah, David S. Reay, and Peter Higgins. 2018. “The Impact of Global Dietary Guidelines on Climate Change.” *Global Environmental Change* 49 (March):46–55. <https://doi.org/10.1016/j.gloenvcha.2018.02.005>.

Shankar, Bhavani, C. S. Srinivasan, and Xavier Irz. 2008. “World Health Organization Dietary Norms: A Quantitative Evaluation of Potential Consumption Impacts in the United States, United Kingdom, and France.” *Review of Agricultural Economics* 30 (1): 151–75. <https://doi.org/10.1111/j.1467-9353.2007.00397.x>.

Springmann, Marco, Keith Wiebe, Daniel Mason-D’Croz, Timothy B Sulser, Mike Rayner, and Peter Scarborough. 2018. “Health and Nutritional Aspects of Sustainable Diet Strategies and Their Association with Environmental Impacts: A Global Modelling Analysis with Country-Level Detail.” *The Lancet Planetary Health* 2 (10): e451–61. <https://doi.org/10.1016/S2542-5196(18)30206-7>.

Wilson, Nick, Nhung Nghiem, Cliona Ni Mhurchu, Helen Eyles, Michael G. Baker, and Tony Blakely. 2013. “Foods and Dietary Patterns That Are Healthy, Low-Cost, and Environmentally Sustainable: A Case Study of Optimization Modeling for New Zealand.” *PLOS ONE* 8 (3): e59648. <https://doi.org/10.1371/journal.pone.0059648>.

**Appendix F – Notes on the theoretical underpinnings of the optimisation model**

Following Irz et al. (2015), we take into account both nutrient- and food-based recommendations, to which we add a budget constraint, and we simulate the impact on individual diets of complying with these recommendations in a Marshallian context, i.e. by keeping income and prices constant. In additional simulations we also include an environmental constraint. Concretely, for each individual, we minimise an objective function that is the sum of the weighted squared deviations between observed and recommended amounts for each food category, so as to limit the taste cost of dietary change.

To account for substitutions between food items, we incorporate own price elasticities and budget shares for each food category into the quadratic programming (QP) formulation. This approach, based on Green et al. (2015), differs from traditional QP methods by explicitly including the price elasticity for each food group, thus allowing the model to adjust consumption patterns in response to price changes. Budget shares are critical because they represent the proportion of household income spent on each food group, influencing consumers' ability to substitute foods when prices change. Including them ensures the model captures both the financial constraints and the preferences that shape real-world consumption decisions. This allows the optimized diet to be realistic, minimizing changes to existing habits while meeting nutritional and environmental objectives.

Our optimisation model accounts for the food preferences of each individual household member since, for each person, the elasticity to budget share ratio is multiplied by the deviation in the consumption of each food group: this means we took into consideration the initial level of consumption for each food – which is specific to each individual – hence individual food preferences were accounted for in that manner.

### **Constraints shared by both models**

The tables in appendix D and E highlight the constraints applied to each model (i.e. CGD or WHO). Below we list the constraints that the two models share:

First, a budget constraint ensured that, in each model, the daily diet cost does not exceed the daily income available to each consumer, thus guaranteeing that the simulated diet is indeed affordable:

$\left( 1 \right) \sum_{i\in F} {P_{i}Q}_{i}^{'}\leq daily income$

where $Q_{i}^{'}$ represents the quantity consumed of food type i in the optimised model, P_i_ is the price of food group i and F is the set of all 19 food groups.

Moreover, we assumed the simulated consumption of each food group to be equal to or greater than zero, since negative amounts consumed are impossible. Green et al. (2015), Shankar et al. (2008), Srinivasan et al. (2006) also use this constraint.

(2) $Q_{i}^{'}\geq0$

**References**

Green, Rosemary, James Milner, Alan D. Dangour, Andy Haines, Zaid Chalabi, Anil Markandya, Joseph Spadaro, and Paul Wilkinson. 2015. “The Potential to Reduce Greenhouse Gas Emissions in the UK through Healthy and Realistic Dietary Change.” *Climatic Change* 129 (1–2): 253–65. <https://doi.org/10.1007/s10584-015-1329-y>.

Irz, Xavier, Pascal Leroy, Vincent Réquillart, and Louis-Georges Soler. 2015. “Economic Assessment of Nutritional Recommendations.” *Journal of Health Economics* 39 (January):188–210. <https://doi.org/10.1016/j.jhealeco.2014.09.002>.

Shankar, Bhavani, C. S. Srinivasan, and Xavier Irz. 2008. “World Health Organization Dietary Norms: A Quantitative Evaluation of Potential Consumption Impacts in the United States, United Kingdom, and France.” *Review of Agricultural Economics* 30 (1): 151–75. <https://doi.org/10.1111/j.1467-9353.2007.00397.x>.

Srinivasan, C.S., Xavier Irz, and Bhavani Shankar. 2006. “An Assessment of the Potential Consumption Impacts of WHO Dietary Norms in OECD Countries.” *Food Policy* 31 (1): 53–77. <https://doi.org/10.1016/j.foodpol.2005.08.002>.

**Appendix G – Additional figures**

**Figure 12.** Percentage change in emissions by income decile, in the WHO and CDG models with no environmental constraints

As revealed in figure 12 above showing simulated shifts in GHGE by income decile, adopting the CDG recommendations would lead to a considerable rise in emissions for the first and second deciles, a moderate increase for the third through the fifth deciles, and would only result in a slight drop in carbon intensity for high-income consumers (belonging to the 8^th^ to the 10^th^ deciles). The WHO diet would translate into a marginal increase in CO_2_ for the poorest consumers (1^st^ decile) but would be followed by a reduction in emissions that would progressively increase, peaking at a 5.05% drop for the 9^th^ decile.

**Fig. 13.** Diet composition (g), optimised male diets imposing a reduction of 10% and 20% in overall GHGE levels

**Fig. 14.** Diet composition (g), optimised female diets imposing a reduction of 10% and 20% in overall GHGE levels

Figures 13 and 14 above display the simulated diet compositions by gender. Both genders consume considerably more cereals and meat under the WHO scenarios, while fruit intakes are significantly lower. Both men and women have higher intakes of dairy and eggs in the CDG diets than in the WHO alternatives.
